# Predation Pressure on Invertebrate Sentinel Prey Depends on Distance to Forest Edge and Seasonality in Kenyan Tea (*Camellia sinensis*) Plantations

**DOI:** 10.3390/insects16090988

**Published:** 2025-09-22

**Authors:** Titus S. Imboma, Alfredo Venturo, Gábor L. Lövei

**Affiliations:** 1 Ornithology Section, National Museums of Kenya, P.O. Box 40-658, Nairobi 00100, Kenya; imbomati911@gmail.com; 2 Department of Ecology, Faculty of Environmental Sciences, Czech University of Life Sciences, CZ-165 00 Prague, Czech Republic; venturo@fzp.czu.cz; 3 Department of Agroecology, Aarhus University, Flakkebjerg Research Centre, DK-4200 Slagelse, Denmark; 4 HUN-REN-UD Anthropocene Ecology Research Group, University of Debrecen, H-4032 Debrecen, Hungary

**Keywords:** natural enemies, artificial prey, conservation biological control, tropical agroecosystems

## Abstract

Tea is a global commodity, and due to its way of preparation pesticide residues cannot be tolerated. This underlines the importance of non-chemical pest control. Kenya is the third largest global tea producer but the potential of natural enemies against tea pests is unknown. We used artificial caterpillars made of non-drying green plasticine to quantify attack rates by predators on tea canopies at different distances from plantation edges and in the nearby native forest during the wet and dry seasons in three tea-growing mountain regions of Kenya, Kericho, Gatamaiyu and Kakamega. The artificial sentinel caterpillars were readily attacked by arthropods, birds, and sporadically by small mammals. During an exposure time of 24 h, the overall attack rate was 25.0%d^−1^. Natural enemy activity was higher in the dry (35.3%d^−1^) than in the wet (16.7%d^−1^) season. The highest predation pressure was measured in the native forest (41.7%d^−1^) and gradually decreased with increasing distances into the tea plantation (to 8.3%d^−1^ at 40 m from the edge). The rate of decrease from the forest edge towards the centre of the tea plantation was steeper for birds than for arthropods, indicating that birds were more reluctant to use this habitat. The potential for natural pest control in Kenyan tea plantations is high, but planting native trees is recommended especially if bird activity inside tea plantations is to be encouraged.

## Simple Summary

Pesticide residues on tea are highly undesirable, underlining the importance of non-chemical methods to reduce pest densities in this crop. The potential of natural enemies to reduce pest densities in tea is unknown, especially in Kenya, where tea cultivation started only 120 years ago. We used artificial caterpillars to quantify potential predation pressure by natural enemies in tea plantations vs. nearby forest habitats in three tea-growing regions in Kenya, in both the dry and the wet seasons. Most attacks were by arthropods and birds, with sporadic small mammal activity. Attack rates by both arthropods and birds were highest in the native forest. As the distance from the forest edge increased, attack rates on artificial prey decreased, gradually by arthropods and precipitously by birds. Attack rates by birds did not differ between seasons, but arthropod attacks were significantly lower during the wet season. The potential for natural pest control in Kenya is high, but agroforestry using native trees is recommended, especially if the presence and activity of birds on tea plantations are to be encouraged.

## 1. Introduction

Natural ecological processes play a key role in maintaining healthy and functional ecosystems that are the basis of ecosystem services (ESs), from which humans derive numerous benefits [1,2]. One of these benefits is natural pest control: naturally occurring predators and parasitoids (natural enemies, hereafter NEs) reducing pest populations to tolerable levels. Providing favourable conditions for such NEs through conservation biological control (CBC) can increase the efficiency of this ES [3]. The potential for effective CBC is higher in perennial than annual cultivations [4] because the former are long-term agricultural habitats, typically with less drastic and less frequent management interventions that favour the development and maintenance of NE communities. Such NEs have documented beneficial effects by lowering pest densities and improving yield and/or its quality in several tropical perennial cultivations, including coffee (*Coffea arabica*) [5] and cocoa (*Theobroma cacao*) [6].

Tea (*Camellia sinensis*), while native to Asia, is an important perennial crop cultivated in over 54 countries around the world in latitudes between 41° N and 16° S [7]. Tea was originally an understory forest tree and has a long history as a human beverage [7]. Globally, more than 100 species of insect pests have been found to attack tea plants, explaining the use of pesticides in pest management [8,9]. However, as tea cannot be washed before consumption, pesticide residues are undesirable, creating pressure to employ natural, non-chemical pest control methods as much as possible. In such situations, the application of CBC is a valuable management option [3].

Although the first tea plantations were established in Kenya only in the early 20th century [9], the country today is the third largest tea producer in the world, with a total yield of 432.4 million kg y^−1^. Small-scale farmers account for 60% of the total tea production [9]. In Kenya, tea-plantations were established in areas covered by biodiverse Afromontane forests, and today the landscape is a mosaic of forest patches, perennial cultivation, and human settlements [9]. The natural factors supporting economic activities are important as elsewhere in the Global South [10] and should be encouraged by conserving habitats that support CBC. Non-cultivated habitat fragments generally support NE diversity, resulting in increased pest control service [3]. However, this conclusion mostly comes from studies in temperate landscapes containing crops with a long cultivation history (but see [11]), and it is uncertain if the same holds for more recent cultivations. One such crop is tea grown in East Africa, and indeed, we do not know what the potential natural enemy (NE) pressure is on pests in the tea-producing landscapes in that part of the world. To start filling this knowledge gap, we conducted experiments to quantify predation pressure by different potential predators in tea plantations and the adjacent natural forests in three major tea-producing areas of Kenya.

We used the sentinel prey approach with artificial caterpillars, as suggested by Howe et al. [12]. While it is recognised that this only registers the activity of certain sub-groups of NEs, it is a simple and useful comparative method to indicate relative differences in predation pressure [13]. Using artificial caterpillars allowed us to examine the relationship between the intensity of predation pressure in forest areas, as well as on tea plantations at various distances from the forest edge during the dry vs. wet seasons. Specifically, we evaluated the following hypotheses:

H1: *The highest predation pressure would be registered in the original, intact forest areas.* We hypothesised this because we assumed that the species-rich forests offer rich resources for their native inhabitants, and only some of the forest-inhabiting NEs would enter the tea plantations. Additionally, the plantations themselves, being recent additions to the landscape, would not attract non-forest predators that can compensate for the missing forest-bound ones.

H2: *Predation pressure in the forest fragments and the tea plantations will be lower than at the forest edge.* The edge as a transition zone has increased potential for complementary resources from the adjacent habitats [14], which will increase the density of NEs [15]. The presence of edge-specific and non-edge species [16,17] would increase predator diversity and create higher predation pressure.

H3: *Predation pressure by NEs will decrease with increasing distance from the fragment edge.* This could occur due to anthropogenic activities within the matrix and the limited dispersal of NEs from the forested source habitat.

H4: *Predation pressure by birds at increasing distances from the nearest forest fragments will not decrease, whereas predation by invertebrates will decrease more drastically.* This can be expected due to the different mobility of these groups, and this was indeed found in a parallel study in China [18].

H5: *Seasonality will influence predation pressure, but this effect will be lower for vertebrates than for invertebrates.* Seasonality in the tropics is often driven by seasonality in precipitation, and the effects of dry vs. wet seasons are higher for ectotherms such as arthropods than homoiothermic vertebrates [19].

Our results partially supported these hypotheses: the highest predation pressure was registered in the forest areas (H1 supported) and decreased at increasing distances from the forest edge (H3 supported). Seasonality was also different as predicted (H5 supported). However, predation pressure was not highest at the edge (H2 not supported), and bird attack rates rapidly decreased towards the tea plantation interiors (H4 not supported).

## 2. Materials and Methods

### 2.1. Study Area

The three study locations in Kenya included the Gatamaiyu forest (00.97° S, 37.00° E, 2300 m a.s.l.) on the slopes of the Aberdares mountain ranges the Kericho/Mau forest in the Mau forest complex (00.40° S, 35.00° E, 2155 m a.s.l.) and the Kakamega tropical rain forest (00.24° N, 34.54° E, 1622 m a.s.l.). All three are in the Afromontane forest region. The selected tea plantations always shared a border with a native forest or suitable-sized forest patch. Tea pruning was similar on all plantations, to a height of ca. 1.2 m that allows pickers to work comfortably.

#### 2.1.1. Gatamaiyu

The Gatamaiyu forest, part of the Kikuyu escarpment, about 40 km from Nairobi, is a high-altitude forest on the lower parts of the Aberdares ranges. It covers a total area of 37,619 ha, with 500 ha of tea plantations. Dominant tree species near the edge, included *Neoboutonia macrocalyx* and *Macaranga capensis*, *Ocotea usambarensis*, *Aningeria adolfi-friendrici* and *Syzygium guineense*. About 20 m from the edge, the forest became more undisturbed, with dominant trees of the cedar (*Juniperus procera*), *Podocarpus latifolius* and the olives *Olea europea*, *O. capensis* and *O. hochstetteri*. The plantations studied (Figure S1a) were conventionally managed. Caterpillars were exposed on 5–7 February 2018 (wet season) and 18–20 October 2019 (dry season).

#### 2.1.2. Kericho

In Kericho, studies were conducted on the James Finlays Kenya (JFK) farm along the Mau Forest complex. This is among the larger private tea farms operating in Kericho, which lies in southwest Kenya on the eastern part of the Great Rift Valley. The JFK operates on a total area of 5908 ha of both conventional and organic tea farming, with 1500 ha of indigenous forest and 2500 ha of exotic eucalyptus forest. The dominant indigenous trees included *A. adolfi-friedericii*, *Strombosia scheffleri*, *Polyscias kikuyuensis*, *O. capensis*, *Prunus africana*, *Albizia gummifera* and *P. latifolius*. The study was carried out on one organic and one conventional tea plantation, while the forest sites were in the neighbouring continuous indigenous Mau Forest block and a riparian forest fragment along a local river (Figure S1b). Caterpillars were exposed on 22–24 February 2018 (wet season) and 2–4 September 2019 (dry season).

#### 2.1.3. Kakamega

The Kakamega forest covers a total area of 14,820 ha, with 11,279 ha of natural forest, 1604 ha of plantation forest and 431 ha of tea plantations. The forest is fragmented by roads running through most parts and along the tea plantation—forest edge. Dominant tree species at our study sites (Figure S1c) included *Pr. africana*, *O. capensis*, *Maesopsis emini*, *Croton megalocarpus*, *Funtumia latifolia* and *Markhamia lutea*. All the tea plantations were under conventional management. Caterpillars were exposed on 9–11 February 2018 (wet season) and 9–11 October 2019 (dry season).

### 2.2. Predation Pressure on Artificial Caterpillars

We assessed predation pressure on the tea canopy and the low canopies of the forest interior using the sentinel prey approach [12]. Green artificial caterpillars (22 mm long, 3 mm in diameter) made of non-drying green plasticine (Staedtler, Nuernberg, Germany) were used. At each of the three study locations, two sites were laid out, at least 300 m from each other. At each site, five positions were selected: inside a forest (>30 m from the edge), at the edge, and at 5 m, 20 m, and 40 m from the edge into the tea plantation. At each position, 10 caterpillars were glued on a leaf of a tea plant or a suitable bush in the forest using superglue (cyanoacrylate adhesive), amounting to a total of 300 caterpillars per season. To minimise the potential effect of the solvent, caterpillars were fixed with only a small drop of glue. Individual caterpillars were 5 m from each other, and the line of caterpillars was running parallel to the forest edge. Thus, a total of 100 caterpillars were used in each site at each season. The artificial prey were left on site for 24 h before they were observed for signs of predation, photographed and carefully removed for further identification in the laboratory using a desktop magnifier (10× magnification). To avoid damage during transportation, each dummy caterpillar was assigned a number and stored in an individual Eppendorf tube. Predators were identified from their characteristic mandible, beak, or tooth marks [12]. In order not to inflate predation intensity, the occasional multiple attack marks, if caused by the same type of predator, were scored as a single attack. Two sessions were performed at each site, one during the wet (February/March) and a second one during the dry (September/October) season, always using the same positions. A total of 600 artificial caterpillars were used for the experiments during the two seasons.

### 2.3. Data Analysis

We analysed the data using the statistical software R version 4.1.1 [20] with the packages *MuMIn* [21] and *lsmeans* [22]. Attack rates on artificial caterpillars by all predators, arthropods and birds were analyzed through generalized linear mixed-effect models (GLMMs) using a binomial error distribution. For total predation (all predators combined), we used GLMMs that included region and field as random effects to account for the nested hierarchical spatial structure. Following Bolker [23], we conducted standard GLMM diagnostics: plots of Pearson residuals versus fitted values and covariates, checks for influential observations, and an overdispersion test (Pearson χ^2^ over residual d.f.). We also evaluated singularity (zero estimated RE variances or |ρ| ≈ 1) and compared simpler random-effects structures by AIC. For taxon-specific subsets, GLMMs with random intercepts for field (nested in region) frequently failed to converge or were singular, indicating insufficient information to estimate among-cluster variance. Remedies targeting extra-binomial variation (beta-binomial likelihood in glmmTMB) and an observation-level random effect, along with centred predictors and the “bobyqa” optimiser with increased iterations, did not resolve these diagnostics. We inspected conditional modes (q–q plots) to rule out group-level outliers driving misfit. Given this evidence, we analysed taxon-specific predation with simpler GLMs, treating the field site as a fixed effect. Full initial models included the fixed effects of region (Gatamaiyu, Kericho or Kakamega), habitat (forest interior, forest edge, tea plantation at 5 m, 20 m, and 40 m from the forest edge), sampling period (wet or dry season), and the interaction between habitat and sampling period, while site (2 sites at each location) was considered a random factor. Model selection was performed using the dredge function (MuMIn package), evaluating all possible combinations of predictors. Given the relatively small sample sizes and to avoid overfitting, we first used a model selection approach to identify predictors of importance: only models with ΔAICc ≤ 2 [24] from the best-fitting model were considered competitive and subsequently averaged. Field identity was intentionally kept for all GLMs models to explicitly account for differences among fields. Model averaging thus provided robust parameter estimates and reduced bias from model uncertainty. We report results only for predictors included in the averaged models (Supplementary Table S1). Differences among levels of significant predictors and their interactions were further explored using pairwise post hoc multiple comparisons through estimated marginal means (EMMs) with Bonferroni correction for multiple testing [25] (Supplementary Tables S2 and S3).

## 3. Results

### 3.1. General Predation Pressure

A total of 159 caterpillars were attacked with 162 predation marks (3 caterpillars at Kakamega were attacked by a bird as well as an arthropod), amounting to a total attack rate of 27.0%d^−1^. Overall attack rates were nearly equal between birds and arthropods at all three locations (Table 1). Mammal predation was observed in Kericho/Mau (4.5%d^−1^), with a single attack in Kakamega and none in Gatamaiyu. Forest habitats witnessed the highest (41.7%d^−1^) attack rates at all the sites, followed by the forest edge (36.7%d^−1^). Averaged models revealed significant effects of position, season, and region on total attack rates. Specifically, total attack rates decreased with increasing distance from the forest during the wet season, while rates were higher in Mau/Kericho. Additionally, a significant interaction indicated a reduction in attack rates during the wet season in Mau/Kericho compared to Gatamaiyu (Table 2). Significant pairwise differences confirmed that attack rates during the dry season in Mau/Kericho were higher than in Gatamaiyu and Kakamega (Supplementary Table S2).

### 3.2. Seasonal Variation

**Figure 1:**
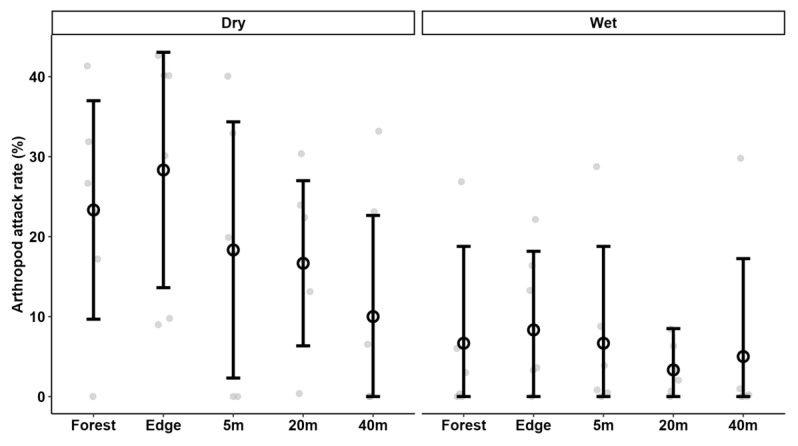
Attack rates by arthropods on artificial caterpillars in three tea-growing areas of Kenya, at increasing distances from the neighbouring native forest in the dry and wet seasons. Data points are means; vertical lines indicate ± one S.D. Grey dots are the attack rates on groups of 10 caterpillars at the individual locations.

**Figure 2:**
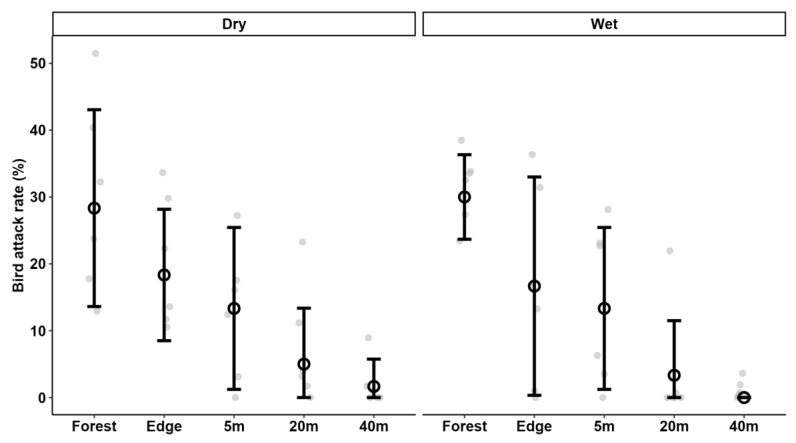
Attack rates by birds on artificial caterpillars in three tea-growing areas of Kenya, at increasing distances from the neighbouring native forest in the dry and wet seasons. Data points are means; vertical lines indicate ± one S.D. Grey dots are the attack rates on groups of 10 caterpillars at the individual location.

Lower predation pressure was observed during the wet (16.7%d^−1^) than the dry (35.3%d^−1^) season but this was driven by differences in arthropod rather than bird predation rates (Table 1, Figure 1 and Figure 2). Seasonality strongly influenced arthropod attack rates, with significantly lower rates observed during the wet than the dry season. Additionally, a significant interaction between region and season indicated that attack rates during the dry season in Mau/Kericho were significantly higher than in Kakamega (Supplementary Table S2). However, seasonality did not affect bird attack rates (Table 2 and Supplementary Table S1).

### 3.3. Predation Pressure in Forest vs. Tea Plantation

Predation pressure sharply decreased with increasing distance from the forest edge into the tea plantation (Table 1 and Table 2), indicating limited spillover of predators from the forest or forest edge. However, this trend was more due to differences in bird predation rates that sharply decreased in both the dry and wet seasons (Figure 2 ) from the forest interior already to 5 m into the tea plantation, becoming even lower further away from the forest. These findings contradicted H4. Arthropod predation pressure was higher at the edge, though this difference was not statistically significant, and was only observed in the dry season (Table 2, Figure 1).

### 3.4. Bird vs. Arthropod Predation Rates

At all three sites, arthropods and birds equally contributed to the overall predation pressure (Table 1). Seasonality strongly influenced arthropod attack rates, with significantly lower rates observed during the wet than the dry season. However, distance from the forest did not appear to significantly affect arthropod attack rates (Table 2, Figure 1).

In contrast, bird attack rates were not affected by seasonality but were significantly influenced by distance from the forest. Bird attack rates markedly declined as the distance from the forest increased, with significant differences observed in all habitats (Table 2 and Supplementary Table S3).

Finally, while regional differences significantly affected total attack rates, these differences were not significant when attack rates were analyzed separately for arthropods and birds. Instead, habitat differences dominated bird attack rates, whereas arthropod attack rates were more strongly influenced by seasonality (Table 1 and Table 2).

## 4. Discussion

This first study on predation pressure in tea plantations in Africa proved that diverse groups of predators associated with natural forests adjacent to tea plantations could use these plantations for foraging. Birds and insects were the most active predators, with some small mammal activity observed in the organically managed plantations at Kericho. In other studies, insectivorous birds and arthropods are also identified as major predators, preying on different herbivorous insects [3,26]. The between-site differences are not unexpected because the three sites were 130–350 km from each other.

In this work, arthropods were more active as NEs in the tea plantations, while bird attacks were highest inside the forest. Predation by birds only extended to the near-edge areas of the tea plantation. Seasonal predation pressure was rather different for arthropods but not for birds. Given that in tropical climates, arthropod density is much higher during the rainy period with massive leaf production [27,28], arthropod predators may have been less hungry than during the dry season, so they were less attracted to the artificial prey. It is also plausible that arthropod activity stopped during rains and did not immediately restart after the rain stopped, so the activity level in general could also be lower. This did not hold for birds: invertivores usually breed during the rainy season [29,30], and have to feed not only themselves but their chicks as well. The presence of newly fledged, inexperienced birds would also increase attack rates on the artificial caterpillars.

### 4.1. Method Limitations

Our quantification of predation pressure is conservative because the artificial caterpillars are attacked by only a subgroup of NEs. The artificial caterpillars neither move nor transmit chemical signals, so NEs that depend on such cues will only accidentally find and attack such artificial prey. For example, parasitoid attacks on artificial caterpillars are only occasionally registered [26], although they are important NEs of Lepidoptera. The effects of pathogens are not registered either, although they are also important mortality factors [31]. Consequently, the recorded attack rates underestimated the overall pressure by NEs that real caterpillars or other invertebrate pests may experience. Also, an increase in attack rates can result from either an increased abundance of predators or a change in their activity. Notwithstanding these limitations, the method is suitable for within-region comparisons [13]. The registered difference between attack rates in the different sub-habitats indicated that a more diverse habitat would benefit natural pest control.

### 4.2. Comparison with Other Studies in Tea Plantations

The only comparable study was made in Fujian, southern China [18]. In a similarly structured landscape and identical experimental design, the predation pressure on artificial caterpillars in tea plantations is 21.5–23.0%d^−1^, with lower attack rates in spring than during summer [18]. This is lower than in Kenya, but the seasonality-induced differences were similar (South China is subject to subtropical weather with similar differences in precipitation patterns). Seasonality in tropical and subtropical climates is often driven by precipitation and not temperature differences [19], and the activity patterns of invertebrates are more influenced by precipitation than those of vertebrates.

The main predator groups were also identical in the two countries. However, birds visit tea plantations in China, where bird attack rates do not decrease precipitously as the distance from forest edges increases [18]. This was not so in Kenya, where attack rates by birds decreased sharply as the distance from the forest edge increased. Curiously, bird species richness in tea plantations was only a little lower than in the forest (T.S.Imboma, unpublished). A possible explanation is that tea is a native plant in China, and the local avifauna has adapted to utilise this plant even when planted as a monoculture. In Kenya, tea has an evolutionarily short presence, where British colonialists established tea plantations only in the early 20th century [9]. Apparently, the birds of the African forest are still reluctant to utilize this habitat or do not have the search image of caterpillars there.

### 4.3. Predation Pressure on Artificial Caterpillars in Africa

Although the proof-of-concept experiments of the sentinel prey method used in our experiments were performed in East Africa [12], most subsequent studies using the method originate from the temperate regions of the world [26]. Additionally, the varying length of exposure of the sentinels [26] poses further difficulties for making comparisons. Similarly to our findings, non-crop habitats have higher predation pressure than adjacent cultivated fields in Uganda [32] as well as Madagascar [33]. The presence of trees also increased attack rates in cultivated habitats in Ethiopia [34]. The registered attack rates vary widely, from 58.9%d^−1^ in conservation cotton in Benin [35] to 2.5%d^−1^ in forest in Mauritius [36]. Globally, the median invertebrate predation rate on vegetation is much lower (at 2.9%d^−1^, [26]) than what was registered at our Kenyan sites, indicating the high potential of NEs to reduce arthropod pests in East African tea plantations.

## 5. Conclusions

Our results document that tea plantations, despite their evolutionarily short presence in Kenya, provide habitat to various NEs and that they, in turn, may provide a useful service in terms of pest control. Predation pressure was higher in the dry than the wet season, and at an average of 25%d^−1^, was comparable to values found in Asian habitats where tea is a native plant. The most active predators were arthropods at all three locations. Predator activity was highest in the original, neighbouring forest habitat, and declined as distance from the forest edge increased. Predation by birds declined more sharply than by arthropods with increasing distance from the forest boundary, which differs from what is found in China. Forest birds in Kenya still avoid feeding in tea plantations. If tea production aims to rely more on CBC, agroforestry may be considered, but native trees instead of exotics should be used. The current practice of using exotic tree species such as the Australian *Grevillea robusta* (T.S. Imboma, pers. obs.) may be suboptimal for this purpose.

## Figures and Tables

**Table 1:** Summary statistics of the attack rates on artificial caterpillars placed on vegetation in three tea-growing regions in Kenya. Data are means ± S.D. (n).

Location/Season/Habitat	Attack Rates (%d^−1^) by		
	All Predators	Arthropods	Birds
Gatamaiyu	25.0 ± 16.7 (20)	12.5 ± 15.5 (20)	12.5 ± 11.6 (20)
Kericho/Mau	31.5 ± 27.4 (20)	14.0 ± 14.3 (20)	15.0 ± 14.7 (20)
Kakamega	21.5 ± 19.0 (20)	11.5 ± 12.3 (20)	11.5 ± 15.7 (20)
Wet season	16.7 ± 15.4 (30)	6.0 ± 10.0 (30)	12.7 ± 14.4 (30)
Dry season	35.3 ± 23.0 (30)	19.3 ± 14.1 (30)	13.3 ± 13.7 (30)
Forest	41.7 ± 14.0 (12)	15.0 ± 15.1 (12)	29.2 ± 10.8 (12)
Forest edge	36.7 ± 25.7 (12)	18.3 ± 15.9 (12)	17.5 ± 12.9 (12)
Tea plantation, 5 m	26.7 ± 21.9 (12)	12.5 ± 14.8 (12)	13.3 ± 11.5 (12)
Tea plantation, 20 m	16.7 ± 13.0 (12)	10.0 ± 10.4 (12)	4.2 ± 7.9 (12)
Tea plantation, 40 m	8.3 ± 12.7 (12)	7.5 ± 12.2 (12)	0.8 ± 2.9 (12)

**Table 2:** Summary of fixed effects included in the final models for each response variable: total predation, insect predation, and bird predation. Each column represents a separate binomial GLMM. Models are based on the model selection results presented in Supplementary Table S1. The table shows the direction of the effect of each predictor relative to the baseline levels: Forest (Location), Gatamaiyu (Region), and Dry season (Season), which are included in the intercept. Arrows indicate the direction of the relationship: (→) no significant effect, (↗) significant positive effect, (↘) significant negative effect. A dash (–) indicates that the predictor or interaction term was not included in the final model after model selection (based on AICc).

Predictors	Responses		
	Total Predation	Insect Predation	Bird Predation
(Intercept)	→	↘	↘
Edge	→	→	↘
Near edge	↘	→	↘
Outer center	↘	→	↘
Center	↘	→	↘
Kakamega	→	–	–
Mau/Kericho	↗	–	–
Wet season	↘	↘	–
Kakamega—Wet season	→	–	–
Mau/Kericho—Wet season	↘	–	–

## Data Availability

The original data on caterpillar attacks are available in Supplementary Table S4.

## Supplementary Materials

The following supporting information can be downloaded at: https://www.mdpi.com/article/10.3390/insects16090988/s1, Figure S1: Maps of the study sites at Gatamaiyu (a), Kericho (b) and Kakamega (c). Symbols indicate the centroids of the study locations; Table S1: Summary of predictor inclusion across averaged models for each response variable based on Akaike Information Criterion corrected for small sample size (AICc). Models were selected using the dredge function in R, and only those within ΔAICc < 2 of the top model were included in the model averaging. A “+” indicates that the predictor or interaction term was included in the averaged model for the given response variable, while “–” indicates it was not; Table S2: Pairwise comparisons of estimated marginal means (EMMs) for all categorical predictors and interactions with more than two levels, based on the total predation averaged model shown in Table S1. Results are derived using the emmeans package in R with Bonferroni-adjusted *p*-values for multiple comparisons. Reported values include the estimated difference in log-odds (Estimate), standard error (SE), and adjusted *p*-value for each contrast. Only predictors and interactions identified as significant in the model selection process were included in this analysis; Table S3: Pairwise comparisons of estimated marginal means (EMMs) for all categorical predictors and interactions with more than two levels, based on the bird predation averaged model shown in Supplementary Table S1. Results are derived using the *emmeans* package in R with Bonferroni-adjusted *p*-values for multiple comparisons. Reported values include the estimated difference in log-odds (Estimate), standard error (SE), and adjusted *p*-value for each contrast. Only predictors and interactions identified as significant in the model selection process were included in this analysis; Table S4: Data on predator attacks on individual artificial caterpillars in three Kenyan tea-growing regions, Gatamaiyu, Kericho and Kakamega.
