# Supplementary material for: Predation Pressure on Invertebrate Sentinel Prey Depends on Distance to Forest Edge and Seasonality in Kenyan Tea (Camellia sinensis) Plantations
Source: Insects. 2025 Sep 22;16(9):988. doi: 10.3390/insects16090988 (PMC12471070; doi:10.3390/insects16090988)
Supplement: Supplementary file 1 [file insects-16-00988-s001.zip › insects-3835212-supplementary.pdf]

Supplementary material to the paper

Predation pressure on invertebrate sentinel prey depends on distance to forest edge and seasonality in Kenyan tea (*Camellia sinensis*) plantations.

By Titus S. Imboma [1], Alfredo Venturo[2] and Gábor L. Lövei [3,4*]

[1] Ornithology Section, National Museums of Kenya, P.O.Box 40-658 Nairobi, Kenya; e-amil: imbomati911@gmail.com
[2] Department of Ecology, Faculty of Environmental Sciences, Czech University of Life Sciences, CZ-165 00 Prague - Suchdol, Czech Republic - email: venturo@fzp.czu.cz
[3] Department of Agroecology, Aarhus University, Flakkebjerg Research Centre, DK-4200 Slagelse, Denmark; e-mail: gabor.lovei@agro.au.dk
[4] HUN-REN-DE Anthropocene Ecology Research Group, Debrecen University, DK-4032 Debrecen, Hungary
* Correspondence: gabor.lovei@agro.au.dk

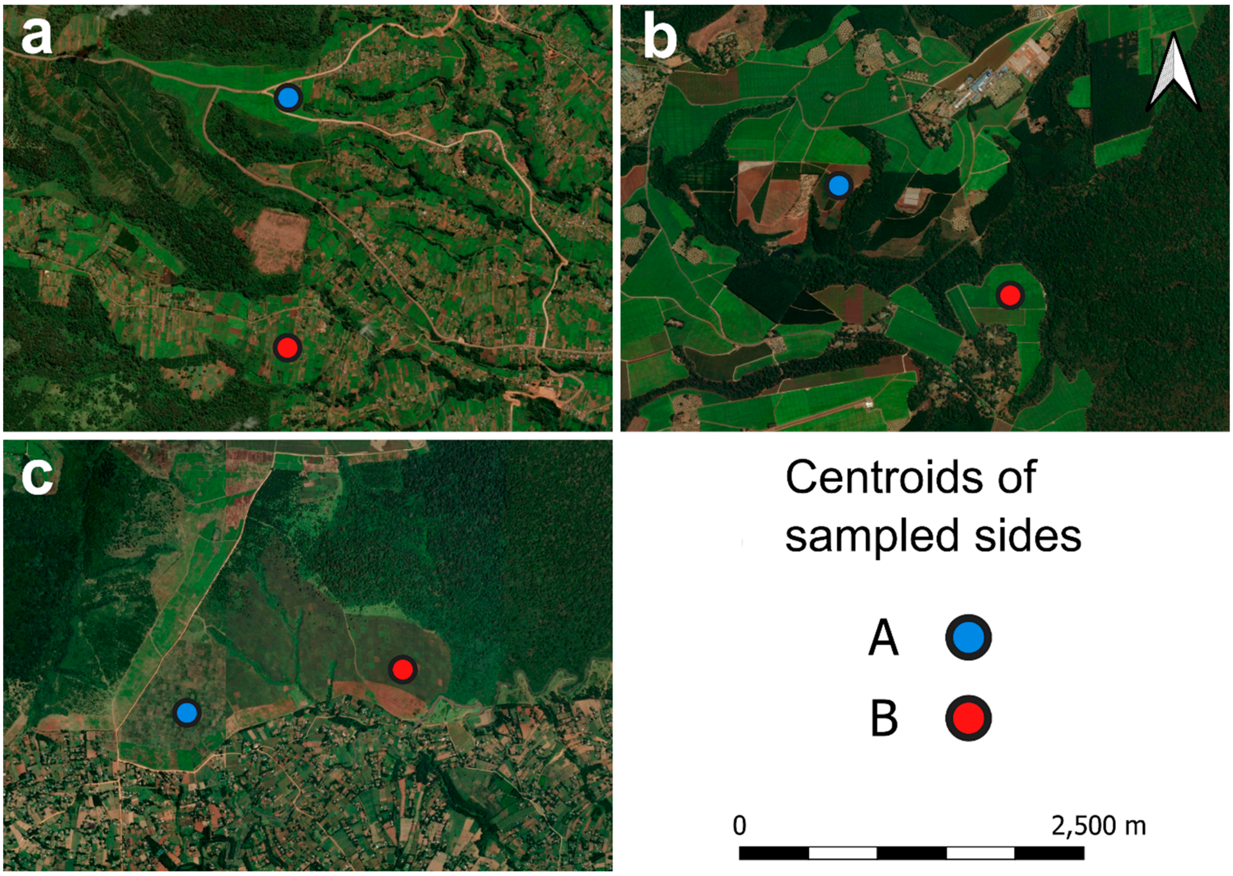

Supplementary Figure S1. Maps of the study sites at Gatamaiyu (a), Kericho (b) and Kakamega (c). Symbols indicate the centroids of the study locations**.**

Supplementary Table S1. Summary of predictor inclusion across averaged models for each response variable based on Akaike Information Criterion corrected for small sample size (AICc). Models were selected using the dredge function in R, and only those within ΔAICc < 2 of the top model were included in the model averaging. A "+" indicates that the predictor or interaction term was included in the averaged model for the given response variable, while "–" indicates it was not.

Predictors	Responses		
	Total predation	Insect predation	Bird predation
Location	+	+	+
Region	+	-	-
Season	+	+	-
Location:Region	-	-	-
Location:Season	-	-	-
Region:Season	+	-	-

Supplementary Table S2. Pairwise comparisons of estimated marginal means (EMMs) for all categorical predictors and interactions with more than two levels, based on the total predation averaged model shown in Supplementary Table 1. Results are derived using the emmeans package in R with Bonferroni-adjusted p-values for multiple comparisons. Reported values include the estimated difference in log-odds (Estimate), standard error (SE), and adjusted p-value for each contrast. Only predictors and interactions identified as significant in the model selection process were included in this analysis.

**Contrast**	**Estimate**	**Standard error**	**P-value**
Forest vs. Edge	0.233	0.279	1.000
Forest vs. Near edge	0.747	0.292	0.615
Forest vs. Outer center	1.398	0.323	**0.001**
Forest vs. Center	2.234	0.394	**0.000**
Edge vs. Near edge	0.514	0.295	1.000
Edge vs. Outer center	1.165	0.325	**0.019**
Edge vs. Center	2.001	0.395	**0.000**
Near edge vs. Outer center	0.651	0.334	1.000
Near edge vs. Center	1.488	0.403	**0.013**
Outer center vs. Center	0.836	0.423	1.000
Gatamaiyu vs. Kakamega	0.209	0.272	1.000
Gatamaiyu vs. Mau/Kericho	-0.239	0.273	1.000
Kakamega vs. Mau/Kericho	-0.448	0.278	1.000
Gatamaiyu dry vs. Kakamega dry	0.273	0.346	1.000
Gatamaiyu dry vs. Mau/Kericho dry	-0.867	0.331	0.247
Gatamaiyu dry vs. Gatamaiyu wet	0.713	0.350	1.000
Gatamaiyu dry vs. Kakamega wet	0.858	0.372	0.592
Gatamaiyu dry vs. Mau/Kericho wet	1.101	0.388	0.127
Kakamega dry vs. Mau/Kericho dry	-1.140	0.339	**0.022**
Kakamega dry vs. Gatamaiyu wet	0.440	0.371	1.000
Kakamega dry vs. Kakamega wet	0.585	0.365	1.000
Kakamega dry vs. Mau/Kericho wet	0.829	0.394	0.994
Mau/Kericho dry vs. Gatamaiyu wet	1.580	0.359	**0.000**
Mau/Kericho dry vs. Kakamega wet	1.725	0.367	**0.000**
Mau/Kericho dry vs. Mau/Kericho wet	1.969	0.370	**0.000**
Gatamaiyu wet vs. Kakamega wet	0.145	0.395	1.000
Gatamaiyu wet vs. Mau/Kericho wet	0.389	0.410	1.000
Kakamega wet vs. Mau/Kericho wet	0.244	0.417	1.000

Supplementary Table S3. Pairwise comparisons of estimated marginal means (EMMs) for all categorical predictors and interactions with more than two levels, based on the bird predation averaged model shown in Supplementary Table S1. Results are derived using the emmeans package in R with Bonferroni-adjusted p-values for multiple comparisons. Reported values include the estimated difference in log-odds (Estimate), standard error (SE), and adjusted p-value for each contrast. Only predictors and interactions identified as significant in the model selection process were included in this analysis.

Contrast	Estimate	Standard error	P-value
Forest vs. Edge	0.666	0.314	0.338
Forest vs. Near edge	0.989	0.336	**0.033**
Forest vs. Outer center	2.255	0.500	**0.000**
Forest vs. Center	3.899	1.024	**0.001**
Edge vs. Near edge	0.322	0.361	1.000
Edge vs. Outer center	1.588	0.517	**0.021**
Edge vs. Center	3.233	1.033	**0.017**
Near edge vs. Outer center	1.266	0.530	0.170
Near edge vs. Center	2.911	1.040	0.051
Outer center vs. Center	1.644	1.103	1.000

Supplementary Table S4. Data on predator attacks on individual artificial caterpillars in three Kenyan tea-growing regions, Gatamayiu, Kericho and Kakamega.

Year	Date	Season	Location	Habitat	Site	Replicate	Predation	Insects	Birds	Mamals	Unknown	Lost	Distance	Predator
2018	9-Feb-18	wet	Kakamega	Forest	A	1	0	0	0	0	0	0	Forest	
2018	9-Feb-18	wet	Kakamega	Forest	A	2	1	0	1	0	0	0	Forest	Bird
2018	9-Feb-18	wet	Kakamega	Forest	A	3	0	0	0	0	0	0	Forest	
2018	9-Feb-18	wet	Kakamega	Forest	A	4	0	0	0	0	0	0	Forest	
2018	9-Feb-18	wet	Kakamega	Forest	A	5	1	0	1	0	0	0	Forest	Bird
2018	9-Feb-18	wet	Kakamega	Forest	A	6	0	0	0	0	0	0	Forest	
2018	9-Feb-18	wet	Kakamega	Forest	A	7	0	0	0	0	0	0	Forest	
2018	9-Feb-18	wet	Kakamega	Forest	A	8	0	0	0	0	0	0	Forest	
2018	9-Feb-18	wet	Kakamega	Forest	A	9	1	0	1	0	0	0	Forest	Bird
2018	9-Feb-18	wet	Kakamega	Forest	A	10	1	0	1	0	0	0	Forest	Bird
2018	9-Feb-18	wet	Kakamega	Edge	A	1	1	0	1	0	0	0	0	Bird
2018	9-Feb-18	wet	Kakamega	Edge	A	2	1	0	1	0	0	0	0	Bird
2018	9-Feb-18	wet	Kakamega	Edge	A	3	0	0	0	0	0	0	0	
2018	9-Feb-18	wet	Kakamega	Edge	A	4	0	0	0	0	0	0	0	
2018	9-Feb-18	wet	Kakamega	Edge	A	5	1	0	1	0	0	0	0	Bird
2018	9-Feb-18	wet	Kakamega	Edge	A	6	0	0	0	0	0	0	0	
2018	9-Feb-18	wet	Kakamega	Edge	A	7	0	0	0	0	0	0	0	
2018	9-Feb-18	wet	Kakamega	Edge	A	8	0	0	0	0	0	0	0	
2018	9-Feb-18	wet	Kakamega	Edge	A	9	0	0	0	0	0	0	0	
2018	9-Feb-18	wet	Kakamega	Edge	A	10	0	0	0	0	0	0	0	
2018	9-Feb-18	wet	Kakamega	Tea5M	A	1	0	0	0	0	0	0	5	
2018	9-Feb-18	wet	Kakamega	Tea5M	A	2	0	0	0	0	0	0	5	
2018	9-Feb-18	wet	Kakamega	Tea5M	A	3	0	0	0	0	0	0	5	
2018	9-Feb-18	wet	Kakamega	Tea5M	A	4	0	0	0	0	0	0	5	
2018	9-Feb-18	wet	Kakamega	Tea5M	A	5	0	0	0	0	0	0	5	
2018	9-Feb-18	wet	Kakamega	Tea5M	A	6	0	0	0	0	0	0	5	
2018	9-Feb-18	wet	Kakamega	Tea5M	A	7	0	0	0	0	0	0	5	
2018	9-Feb-18	wet	Kakamega	Tea5M	A	8	0	0	0	0	0	0	5	
2018	9-Feb-18	wet	Kakamega	Tea5M	A	9	0	0	0	0	0	0	5	
2018	9-Feb-18	wet	Kakamega	Tea5M	A	10	0	0	0	0	0	0	5	
2018	9-Feb-18	wet	Kakamega	Tea20M	A	1	0	0	0	0	0	0	20	
2018	9-Feb-18	wet	Kakamega	Tea20M	A	2	0	0	0	0	0	0	20	
2018	9-Feb-18	wet	Kakamega	Tea20M	A	3	0	0	0	0	0	0	20	
2018	9-Feb-18	wet	Kakamega	Tea20M	A	4	0	0	0	0	0	0	20	
2018	9-Feb-18	wet	Kakamega	Tea20M	A	5	0	0	0	0	0	0	20	
2018	9-Feb-18	wet	Kakamega	Tea20M	A	6	1	1	0	0	0	0	20	Insect
2018	9-Feb-18	wet	Kakamega	Tea20M	A	7	0	0	0	0	0	0	20	
2018	9-Feb-18	wet	Kakamega	Tea20M	A	8	0	0	0	0	0	0	20	
2018	9-Feb-18	wet	Kakamega	Tea20M	A	9	0	0	0	0	0	0	20	
2018	9-Feb-18	wet	Kakamega	Tea20M	A	10	1	0	0	1	0	0	20	Mammal
2018	9-Feb-18	wet	Kakamega	Tea40M	A	1	0	0	0	0	0	0	40	
2018	9-Feb-18	wet	Kakamega	Tea40M	A	2	0	0	0	0	0	0	40	
2018	9-Feb-18	wet	Kakamega	Tea40M	A	3	0	0	0	0	0	0	40	

2018	9-Feb-18	wet	Kakamega	Tea40M	A	4	0	0	0	0	0	0	40	
2018	9-Feb-18	wet	Kakamega	Tea40M	A	5	0	0	0	0	0	0	40	
2018	9-Feb-18	wet	Kakamega	Tea40M	A	6	0	0	0	0	0	0	40	
2018	9-Feb-18	wet	Kakamega	Tea40M	A	7	0	0	0	0	0	0	40	
2018	9-Feb-18	wet	Kakamega	Tea40M	A	8	0	0	0	0	0	0	40	
2018	9-Feb-18	wet	Kakamega	Tea40M	A	9	0	0	0	0	0	0	40	
2018	9-Feb-18	wet	Kakamega	Tea40M	A	10	0	0	0	0	0	0	40	
2018	10-Feb-18	wet	Kakamega	Forest	B	1	0	0	0	0	0	0	Forest	
2018	10-Feb-18	wet	Kakamega	Forest	B	2	0	0	0	0	0	0	Forest	
2018	10-Feb-18	wet	Kakamega	Forest	B	3	1	0	1	0	0	0	Forest	Bird
2018	10-Feb-18	wet	Kakamega	Forest	B	4	1	1	0	0	0	0	Forest	Inect
2018	10-Feb-18	wet	Kakamega	Forest	B	5	0	0	0	0	0	0	Forest	
2018	10-Feb-18	wet	Kakamega	Forest	B	6	0	0	0	0	0	0	Forest	
2018	10-Feb-18	wet	Kakamega	Forest	B	7	0	0	0	0	0	0	Forest	
2018	10-Feb-18	wet	Kakamega	Forest	B	8	2	1	1	0	0	0	Forest	Bird+insect
2018	10-Feb-18	wet	Kakamega	Forest	B	9	0	0	0	0	0	0	Forest	
2018	10-Feb-18	wet	Kakamega	Forest	B	10	2	1	1	0	0	0	Forest	Bird+insect
2018	10-Feb-18	wet	Kakamega	Edge	B	1	1	0	0	0	1	0	0	Unknown
2018	10-Feb-18	wet	Kakamega	Edge	B	2	0	0	0	0	0	0	0	
2018	10-Feb-18	wet	Kakamega	Edge	B	3	0	0	0	0	0	0	0	
2018	10-Feb-18	wet	Kakamega	Edge	B	4	0	0	1	0	0	0	0	Bird
2018	10-Feb-18	wet	Kakamega	Edge	B	5	0	0	1	0	0	0	0	Bird
2018	10-Feb-18	wet	Kakamega	Edge	B	6	0	0	0	0	0	0	0	
2018	10-Feb-18	wet	Kakamega	Edge	B	7	1	1	0	0	0	0	0	Insect
2018	10-Feb-18	wet	Kakamega	Edge	B	8	2	1	1	0	0	0	0	Bird+insect
2018	10-Feb-18	wet	Kakamega	Edge	B	9	0	0	0	0	0	0	0	
2018	10-Feb-18	wet	Kakamega	Edge	B	10	1	0	1	0	0	0	0	Bird
2018	10-Feb-18	wet	Kakamega	Tea5M	B	1	0	0	0	0	0	0	5	
2018	10-Feb-18	wet	Kakamega	Tea5M	B	2	0	0	0	0	0	0	5	
2018	10-Feb-18	wet	Kakamega	Tea5M	B	3	0	0	0	0	0	0	5	
2018	10-Feb-18	wet	Kakamega	Tea5M	B	4	0	0	0	0	0	0	5	
2018	10-Feb-18	wet	Kakamega	Tea5M	B	5	0	0	0	0	0	0	5	
2018	10-Feb-18	wet	Kakamega	Tea5M	B	6	0	0	0	0	0	0	5	
2018	10-Feb-18	wet	Kakamega	Tea5M	B	7	0	0	0	0	0	0	5	
2018	10-Feb-18	wet	Kakamega	Tea5M	B	8	0	0	0	0	0	0	5	
2018	10-Feb-18	wet	Kakamega	Tea5M	B	9	0	0	0	0	0	0	5	
2018	10-Feb-18	wet	Kakamega	Tea5M	B	10	0	0	0	0	0	0	5	
2018	10-Feb-18	wet	Kakamega	Tea20M	B	1	0	0	0	0	0	0	20	
2018	10-Feb-18	wet	Kakamega	Tea20M	B	2	0	0	0	0	0	0	20	
2018	10-Feb-18	wet	Kakamega	Tea20M	B	3	0	0	0	0	0	0	20	
2018	10-Feb-18	wet	Kakamega	Tea20M	B	4	0	0	0	0	0	0	20	
2018	10-Feb-18	wet	Kakamega	Tea20M	B	5	0	0	0	0	0	0	20	
2018	10-Feb-18	wet	Kakamega	Tea20M	B	6	0	0	0	0	0	0	20	
2018	10-Feb-18	wet	Kakamega	Tea20M	B	7	0	0	0	0	0	0	20	
2018	10-Feb-18	wet	Kakamega	Tea20M	B	8	0	0	0	0	0	0	20	
2018	10-Feb-18	wet	Kakamega	Tea20M	B	9	0	0	0	0	0	0	20	

2018	10-Feb-18	wet	Kakamega	Tea20M	B	10	0	0	0	0	0	0	20	
2018	10-Feb-18	wet	Kakamega	Tea40M	B	1	0	0	0	0	0	0	40	
2018	10-Feb-18	wet	Kakamega	Tea40M	B	2	0	0	0	0	0	0	40	
2018	10-Feb-18	wet	Kakamega	Tea40M	B	3	0	0	0	0	0	0	40	
2018	10-Feb-18	wet	Kakamega	Tea40M	B	4	0	0	0	0	0	0	40	
2018	10-Feb-18	wet	Kakamega	Tea40M	B	5	0	0	0	0	0	0	40	
2018	10-Feb-18	wet	Kakamega	Tea40M	B	6	0	0	0	0	0	0	40	
2018	10-Feb-18	wet	Kakamega	Tea40M	B	7	0	0	0	0	0	0	40	
2018	10-Feb-18	wet	Kakamega	Tea40M	B	8	0	0	0	0	0	0	40	
2018	10-Feb-18	wet	Kakamega	Tea40M	B	9	0	0	0	0	0	0	40	
2018	10-Feb-18	wet	Kakamega	Tea40M	B	10	0	0	0	0	0	0	40	
2018	22/Feb/18	wet	Mau/Kericho	Forest	A	1	0	0	0	0	0	0	Forest	
2018	22/Feb/18	wet	Mau/Kericho	Forest	A	2	0	0	0	0	0	0	Forest	
2018	22/Feb/18	wet	Mau/Kericho	Forest	A	3	0	0	1	0	0	0	Forest	Bird
2018	22/Feb/18	wet	Mau/Kericho	Forest	A	4	0	0	0	0	0	0	Forest	
2018	22/Feb/18	wet	Mau/Kericho	Forest	A	5	0	0	0	0	0	0	Forest	
2018	22/Feb/18	wet	Mau/Kericho	Forest	A	6	1	0	1	0	0	0	Forest	Bird
2018	22/Feb/18	wet	Mau/Kericho	Forest	A	7	1	0	1	0	0	0	Forest	Bird
2018	22/Feb/18	wet	Mau/Kericho	Forest	A	8	1	1	0	0	0	0	Forest	Insect
2018	22/Feb/18	wet	Mau/Kericho	Forest	A	9	0	0	0	0	0	0	Forest	
2018	22/Feb/18	wet	Mau/Kericho	Forest	A	10	0	0	0	0	0	0	Forest	
2018	22/Feb/18	wet	Mau/Kericho	Edge	A	1	0	0	0	0	0	0	0	
2018	22/Feb/18	wet	Mau/Kericho	Edge	A	2	0	0	0	0	0	0	0	
2018	22/Feb/18	wet	Mau/Kericho	Edge	A	3	0	0	0	0	0	0	0	
2018	22/Feb/18	wet	Mau/Kericho	Edge	A	4	0	0	0	0	0	0	0	
2018	22/Feb/18	wet	Mau/Kericho	Edge	A	5	0	0	0	0	0	0	0	
2018	22/Feb/18	wet	Mau/Kericho	Edge	A	6	0	0	0	1	0	0	0	Mammal
2018	22/Feb/18	wet	Mau/Kericho	Edge	A	7	0	0	0	0	0	0	0	
2018	22/Feb/18	wet	Mau/Kericho	Edge	A	8	0	0	0	0	0	0	0	
2018	22/Feb/18	wet	Mau/Kericho	Edge	A	9	0	0	0	0	0	0	0	
2018	22/Feb/18	wet	Mau/Kericho	Edge	A	10	0	0	0	0	0	0	0	
2018	22/Feb/18	wet	Mau/Kericho	Tea5M	A	1	0	0	0	0	0	0	5	
2018	22/Feb/18	wet	Mau/Kericho	Tea5M	A	1	0	0	0	0	0	0	5	
2018	22/Feb/18	wet	Mau/Kericho	Tea5M	A	3	0	0	0	0	0	0	5	
2018	22/Feb/18	wet	Mau/Kericho	Tea5M	A	4	0	0	0	0	0	0	5	
2018	22/Feb/18	wet	Mau/Kericho	Tea5M	A	5	0	0	0	0	0	0	5	
2018	22/Feb/18	wet	Mau/Kericho	Tea5M	A	6	0	0	0	0	0	0	5	
2018	22/Feb/18	wet	Mau/Kericho	Tea5M	A	7	0	0	0	0	0	0	5	
2018	22/Feb/18	wet	Mau/Kericho	Tea5M	A	8	0	0	0	0	0	0	5	
2018	22/Feb/18	wet	Mau/Kericho	Tea5M	A	9	1	0	1	0	0	0	5	Bird
2018	22/Feb/18	wet	Mau/Kericho	Tea5M	A	10	1	0	1	0	0	0	5	Bird
2018	22/Feb/18	wet	Mau/Kericho	Tea20M	A	1	0	0	0	0	0	0	20	
2018	22/Feb/18	wet	Mau/Kericho	Tea20M	A	2	0	0	0	0	0	0	20	
2018	22/Feb/18	wet	Mau/Kericho	Tea20M	A	3	0	0	0	0	0	0	20	
2018	22/Feb/18	wet	Mau/Kericho	Tea20M	A	4	0	0	0	0	0	0	20	
2018	22/Feb/18	wet	Mau/Kericho	Tea20M	A	5	0	0	0	0	0	0	20	

2018	22/Feb/18	wet	Mau/Kericho	Tea20M	A	6	0	0	0	0	0	0	20	
2018	22/Feb/18	wet	Mau/Kericho	Tea20M	A	7	0	0	0	0	0	0	20	
2018	22/Feb/18	wet	Mau/Kericho	Tea20M	A	8	0	0	0	0	0	0	20	
2018	22/Feb/18	wet	Mau/Kericho	Tea20M	A	9	0	0	0	0	0	0	20	
2018	22/Feb/18	wet	Mau/Kericho	Tea20M	A	10	0	0	0	0	0	0	20	
2018	22/Feb/18	wet	Mau/Kericho	Tea40M	A	1	0	0	0	0	0	0	40	
2018	22/Feb/18	wet	Mau/Kericho	Tea40M	A	2	0	0	0	0	0	0	40	
2018	22/Feb/18	wet	Mau/Kericho	Tea40M	A	3	0	0	0	0	0	0	40	
2018	22/Feb/18	wet	Mau/Kericho	Tea40M	A	4	0	0	0	0	0	0	40	
2018	22/Feb/18	wet	Mau/Kericho	Tea40M	A	5	0	0	0	0	0	0	40	
2018	22/Feb/18	wet	Mau/Kericho	Tea40M	A	6	0	0	0	0	0	0	40	
2018	22/Feb/18	wet	Mau/Kericho	Tea40M	A	7	0	0	0	0	0	0	40	
2018	22/Feb/18	wet	Mau/Kericho	Tea40M	A	8	0	0	0	0	0	0	40	
2018	22/Feb/18	wet	Mau/Kericho	Tea40M	A	9	0	0	0	0	0	0	40	
2018	22/Feb/18	wet	Mau/Kericho	Tea40M	A	10	0	0	0	0	0	0	40	
2018	23/Feb/18	wet	Mau/Kericho	Forest	B	1	0	0	0	0	0	0	Forest	
2018	23/Feb/18	wet	Mau/Kericho	Forest	B	2	0	0	0	0	0	0	Forest	
2018	23/Feb/18	wet	Mau/Kericho	Forest	B	3	0	0	0	0	0	0	Forest	
2018	23/Feb/18	wet	Mau/Kericho	Forest	B	4	0	0	0	0	0	0	Forest	
2018	23/Feb/18	wet	Mau/Kericho	Forest	B	5	1	0	1	0	0	0	Forest	Bird
2018	23/Feb/18	wet	Mau/Kericho	Forest	B	6	1	0	1	0	0	0	Forest	Bird
2018	23/Feb/18	wet	Mau/Kericho	Forest	B	7	0	0	0	0	0	0	Forest	
2018	23/Feb/18	wet	Mau/Kericho	Forest	B	8	1	0	1	0	0	0	Forest	Bird
2018	23/Feb/18	wet	Mau/Kericho	Forest	B	9	0	0	0	0	0	0	Forest	
2018	23/Feb/18	wet	Mau/Kericho	Forest	B	10	0	0	0	0	0	0	Forest	
2018	23/Feb/18	wet	Mau/Kericho	Edge	B	1	0	0	0	0	0	0	0	
2018	23/Feb/18	wet	Mau/Kericho	Edge	B	2	1	0	1	0	0	0	0	Bird
2018	23/Feb/18	wet	Mau/Kericho	Edge	B	3	1	1	0	0	0	0	0	Insect
2018	23/Feb/18	wet	Mau/Kericho	Edge	B	4	0	0	0	0	0	0	0	
2018	23/Feb/18	wet	Mau/Kericho	Edge	B	5	0	0	0	0	0	0	0	
2018	23/Feb/18	wet	Mau/Kericho	Edge	B	6	0	0	0	1	0	0	0	Mammal
2018	23/Feb/18	wet	Mau/Kericho	Edge	B	7	1	1	0	0	0	0	0	Insect
2018	23/Feb/18	wet	Mau/Kericho	Edge	B	8	0	0	0	0	0	0	0	
2018	23/Feb/18	wet	Mau/Kericho	Edge	B	9	0	0	0	0	0	0	0	
2018	23/Feb/18	wet	Mau/Kericho	Edge	B	10	0	0	0	0	0	0	0	
2018	23/Feb/18	wet	Mau/Kericho	Tea5M	B	1	1	0	1	0	0	0	5	Bird
2018	23/Feb/18	wet	Mau/Kericho	Tea5M	B	2	0	1	0	0	0	0	5	Insect
2018	23/Feb/18	wet	Mau/Kericho	Tea5M	B	3	0	0	0	0	0	0	5	
2018	23/Feb/18	wet	Mau/Kericho	Tea5M	B	4	0	1	0	0	0	0	5	Insect
2018	23/Feb/18	wet	Mau/Kericho	Tea5M	B	5	0	0	0	0	0	0	5	
2018	23/Feb/18	wet	Mau/Kericho	Tea5M	B	6	0	0	0	0	0	0	5	
2018	23/Feb/18	wet	Mau/Kericho	Tea5M	B	7	0	0	0	0	0	0	5	
2018	23/Feb/18	wet	Mau/Kericho	Tea5M	B	8	0	0	0	0	0	0	5	
2018	23/Feb/18	wet	Mau/Kericho	Tea5M	B	9	0	0	0	0	0	0	5	
2018	23/Feb/18	wet	Mau/Kericho	Tea5M	B	10	1	1	0	0	0	0	5	Insect
2018	23/Feb/18	wet	Mau/Kericho	Tea20M	B	1	1	1	0	0	0	0	20	Insect

2018	23/Feb/18	wet	Mau/Kericho	Tea20M	B	2	0	0	0	0	0	0	20	
2018	23/Feb/18	wet	Mau/Kericho	Tea20M	B	3	0	0	0	0	0	0	20	
2018	23/Feb/18	wet	Mau/Kericho	Tea20M	B	4	0	0	0	0	0	0	20	
2018	23/Feb/18	wet	Mau/Kericho	Tea20M	B	5	0	0	0	0	0	0	20	
2018	23/Feb/18	wet	Mau/Kericho	Tea20M	B	6	0	0	0	0	0	0	20	
2018	23/Feb/18	wet	Mau/Kericho	Tea20M	B	7	0	0	0	0	0	0	20	
2018	23/Feb/18	wet	Mau/Kericho	Tea20M	B	8	0	0	0	0	0	0	20	
2018	23/Feb/18	wet	Mau/Kericho	Tea20M	B	9	0	0	0	0	0	0	20	
2018	23/Feb/18	wet	Mau/Kericho	Tea20M	B	10	0	0	0	0	0	0	20	
2018	23/Feb/18	wet	Mau/Kericho	Tea40M	B	1	0	0	0	0	0	0	40	
2018	23/Feb/18	wet	Mau/Kericho	Tea40M	B	2	0	0	0	0	0	0	40	
2018	23/Feb/18	wet	Mau/Kericho	Tea40M	B	3	0	0	0	0	0	0	40	
2018	23/Feb/18	wet	Mau/Kericho	Tea40M	B	4	0	0	0	0	0	0	40	
2018	23/Feb/18	wet	Mau/Kericho	Tea40M	B	5	0	0	0	0	0	0	40	
2018	23/Feb/18	wet	Mau/Kericho	Tea40M	B	6	0	0	0	0	0	0	40	
2018	23/Feb/18	wet	Mau/Kericho	Tea40M	B	7	0	0	0	0	0	0	40	
2018	23/Feb/18	wet	Mau/Kericho	Tea40M	B	8	0	0	0	0	0	0	40	
2018	23/Feb/18	wet	Mau/Kericho	Tea40M	B	9	0	0	0	0	0	0	40	
2018	23/Feb/18	wet	Mau/Kericho	Tea40M	B	10	0	0	0	0	0	0	40	
2018	05/Feb/18	wet	Gatamaiyu	Forest	A	1	1	0	1	0	0	0	Forest	Bird
2018	05/Feb/18	wet	Gatamaiyu	Forest	A	2	0	0	0	0	0	0	Forest	
2018	05/Feb/18	wet	Gatamaiyu	Forest	A	3	0	0	0	0	0	0	Forest	
2018	05/Feb/18	wet	Gatamaiyu	Forest	A	4	0	0	0	0	0	0	Forest	
2018	05/Feb/18	wet	Gatamaiyu	Forest	A	5	1	0	1	0	0	0	Forest	Bird
2018	05/Feb/18	wet	Gatamaiyu	Forest	A	6	0	0	0	0	0	0	Forest	
2018	05/Feb/18	wet	Gatamaiyu	Forest	A	7	0	0	0	0	0	0	Forest	
2018	05/Feb/18	wet	Gatamaiyu	Forest	A	8	0	0	0	0	0	0	Forest	
2018	05/Feb/18	wet	Gatamaiyu	Forest	A	9	0	0	0	0	0	0	Forest	
2018	05/Feb/18	wet	Gatamaiyu	Forest	A	10	1	0	1	0	0	0	Forest	Bird
2018	05/Feb/18	wet	Gatamaiyu	Edge	A	1	0	0	0	0	0	0	0	
2018	05/Feb/18	wet	Gatamaiyu	Edge	A	2	0	0	0	0	0	0	0	
2018	05/Feb/18	wet	Gatamaiyu	Edge	A	3	0	0	0	0	0	0	0	
2018	05/Feb/18	wet	Gatamaiyu	Edge	A	4	0	0	0	0	0	0	0	
2018	05/Feb/18	wet	Gatamaiyu	Edge	A	5	0	0	0	0	0	0	0	
2018	05/Feb/18	wet	Gatamaiyu	Edge	A	6	0	0	0	0	0	0	0	
2018	05/Feb/18	wet	Gatamaiyu	Edge	A	7	0	0	0	0	0	0	0	
2018	05/Feb/18	wet	Gatamaiyu	Edge	A	8	0	0	0	0	0	0	0	
2018	05/Feb/18	wet	Gatamaiyu	Edge	A	9	0	0	0	0	0	0	0	
2018	05/Feb/18	wet	Gatamaiyu	Edge	A	10	0	0	0	0	0	0	0	
2018	05/Feb/18	wet	Gatamaiyu	Tea5M	A	1	1	0	1	0	0	0	5	Bird
2018	05/Feb/18	wet	Gatamaiyu	Tea5M	A	2	0	0	0	0	0	0	5	
2018	05/Feb/18	wet	Gatamaiyu	Tea5M	A	3	0	0	0	0	0	0	5	
2018	05/Feb/18	wet	Gatamaiyu	Tea5M	A	4	0	0	0	0	0	0	5	
2018	05/Feb/18	wet	Gatamaiyu	Tea5M	A	5	1	0	1	0	0	0	5	Bird
2018	05/Feb/18	wet	Gatamaiyu	Tea5M	A	6	0	0	0	0	0	0	5	
2018	05/Feb/18	wet	Gatamaiyu	Tea5M	A	7	0	0	0	0	0	0	5	

2018	05/Feb/18	wet	Gatamaiyu	Tea5M	A	8	0	0	0	0	0	0	5	
2018	05/Feb/18	wet	Gatamaiyu	Tea5M	A	9	0	0	0	0	0	0	5	
2018	05/Feb/18	wet	Gatamaiyu	Tea5M	A	10	0	0	0	0	0	0	5	
2018	05/Feb/18	wet	Gatamaiyu	Tea20M	A	1	0	0	0	0	0	0	20	
2018	05/Feb/18	wet	Gatamaiyu	Tea20M	A	2	0	0	0	0	0	0	20	
2018	05/Feb/18	wet	Gatamaiyu	Tea20M	A	3	0	0	0	0	0	0	20	
2018	05/Feb/18	wet	Gatamaiyu	Tea20M	A	4	0	0	0	0	0	0	20	
2018	05/Feb/18	wet	Gatamaiyu	Tea20M	A	5	0	0	0	0	0	0	20	
2018	05/Feb/18	wet	Gatamaiyu	Tea20M	A	6	0	0	0	0	0	0	20	
2018	05/Feb/18	wet	Gatamaiyu	Tea20M	A	7	0	0	0	0	0	0	20	
2018	05/Feb/18	wet	Gatamaiyu	Tea20M	A	8	0	0	0	0	0	0	20	
2018	05/Feb/18	wet	Gatamaiyu	Tea20M	A	9	0	0	0	0	0	0	20	
2018	05/Feb/18	wet	Gatamaiyu	Tea20M	A	10	0	0	0	0	0	0	20	
2018	05/Feb/18	wet	Gatamaiyu	Tea40M	A	1	0	0	0	0	0	0	40	
2018	05/Feb/18	wet	Gatamaiyu	Tea40M	A	2	0	0	0	0	0	0	40	
2018	05/Feb/18	wet	Gatamaiyu	Tea40M	A	3	0	0	0	0	0	0	40	
2018	05/Feb/18	wet	Gatamaiyu	Tea40M	A	4	0	0	0	0	0	0	40	
2018	05/Feb/18	wet	Gatamaiyu	Tea40M	A	5	0	0	0	0	0	0	40	
2018	05/Feb/18	wet	Gatamaiyu	Tea40M	A	6	0	0	0	0	0	0	40	
2018	05/Feb/18	wet	Gatamaiyu	Tea40M	A	7	0	0	0	0	0	0	40	
2018	05/Feb/18	wet	Gatamaiyu	Tea40M	A	8	0	0	0	0	0	0	40	
2018	05/Feb/18	wet	Gatamaiyu	Tea40M	A	9	0	0	0	0	0	0	40	
2018	05/Feb/18	wet	Gatamaiyu	Tea40M	A	10	0	0	0	0	0	0	40	
2018	06/Feb/18	wet	Gatamaiyu	Forest	B	1	0	0	0	0	0	0	Forest	
2018	06/Feb/18	wet	Gatamaiyu	Forest	B	2	0	0	0	0	0	0	Forest	
2018	06/Feb/18	wet	Gatamaiyu	Forest	B	3	0	0	0	0	0	0	Forest	
2018	06/Feb/18	wet	Gatamaiyu	Forest	B	4	0	0	0	0	0	0	Forest	
2018	06/Feb/18	wet	Gatamaiyu	Forest	B	5	0	0	0	0	0	0	Forest	
2018	06/Feb/18	wet	Gatamaiyu	Forest	B	6	1	0	1	0	0	0	Forest	Bird
2018	06/Feb/18	wet	Gatamaiyu	Forest	B	7	1	0	1	0	0	0	Forest	Bird
2018	06/Feb/18	wet	Gatamaiyu	Forest	B	8	0	0	0	0	0	0	Forest	
2018	06/Feb/18	wet	Gatamaiyu	Forest	B	9	0	0	0	0	0	0	Forest	
2018	06/Feb/18	wet	Gatamaiyu	Forest	B	10	0	0	0	0	0	0	Forest	
2018	06/Feb/18	wet	Gatamaiyu	Edge	B	1	0	0	0	0	0	0	0	
2018	06/Feb/18	wet	Gatamaiyu	Edge	B	2	1	0	1	0	0	0	0	Bird
2018	06/Feb/18	wet	Gatamaiyu	Edge	B	3	0	0	0	0	0	0	0	
2018	06/Feb/18	wet	Gatamaiyu	Edge	B	4	1	0	1	0	0	0	0	Bird
2018	06/Feb/18	wet	Gatamaiyu	Edge	B	5	0	0	0	0	0	0	0	
2018	06/Feb/18	wet	Gatamaiyu	Edge	B	6	0	0	0	0	0	0	0	
2018	06/Feb/18	wet	Gatamaiyu	Edge	B	7	1	1	0	0	0	0	0	Insect
2018	06/Feb/18	wet	Gatamaiyu	Edge	B	8	0	0	0	0	0	0	0	
2018	06/Feb/18	wet	Gatamaiyu	Edge	B	9	0	0	0	0	0	0	0	
2018	06/Feb/18	wet	Gatamaiyu	Edge	B	10	0	0	0	0	0	0	0	
2018	06/Feb/18	wet	Gatamaiyu	Tea5M	B	1	0	0	0	0	0	0	5	
2018	06/Feb/18	wet	Gatamaiyu	Tea5M	B	2	1	1	0	0	0	0	5	Insect
2018	06/Feb/18	wet	Gatamaiyu	Tea5M	B	3	1	0	1	0	0	0	5	Bird

2018	06/Feb/18	wet	Gatamaiyu	Tea5M	B	4	0	0	0	0	0	0	5	
2018	06/Feb/18	wet	Gatamaiyu	Tea5M	B	5	1	0	1	0	0	0	5	Bird
2018	06/Feb/18	wet	Gatamaiyu	Tea5M	B	6	0	0	0	0	0	0	5	
2018	06/Feb/18	wet	Gatamaiyu	Tea5M	B	7	0	0	0	0	0	0	5	
2018	06/Feb/18	wet	Gatamaiyu	Tea5M	B	8	0	0	0	0	0	0	5	
2018	06/Feb/18	wet	Gatamaiyu	Tea5M	B	9	0	0	0	0	0	0	5	
2018	06/Feb/18	wet	Gatamaiyu	Tea5M	B	10	1	0	1	0	0	0	5	Bird
2018	06/Feb/18	wet	Gatamaiyu	Tea20M	B	1	0	0	0	0	0	0	20	
2018	06/Feb/18	wet	Gatamaiyu	Tea20M	B	2	0	0	0	0	0	0	20	
2018	06/Feb/18	wet	Gatamaiyu	Tea20M	B	3	0	0	0	0	0	0	20	
2018	06/Feb/18	wet	Gatamaiyu	Tea20M	B	4	0	0	0	0	0	0	20	
2018	06/Feb/18	wet	Gatamaiyu	Tea20M	B	5	0	0	0	0	0	0	20	
2018	06/Feb/18	wet	Gatamaiyu	Tea20M	B	6	0	0	0	0	0	0	20	
2018	06/Feb/18	wet	Gatamaiyu	Tea20M	B	7	0	0	0	0	0	0	20	
2018	06/Feb/18	wet	Gatamaiyu	Tea20M	B	8	0	0	0	0	0	0	20	
2018	06/Feb/18	wet	Gatamaiyu	Tea20M	B	9	1	0	1	0	0	0	20	Bird
2018	06/Feb/18	wet	Gatamaiyu	Tea20M	B	10	1	0	1	0	0	0	20	Bird
2018	06/Feb/18	wet	Gatamaiyu	Tea40M	B	1	0	0	0	0	0	0	40	
2018	06/Feb/18	wet	Gatamaiyu	Tea40M	B	2	1	1	0	0	0	0	40	Insect
2018	06/Feb/18	wet	Gatamaiyu	Tea40M	B	3	1	1	0	0	0	0	40	Insect
2018	06/Feb/18	wet	Gatamaiyu	Tea40M	B	4	1	1	0	0	0	0	40	Insect
2018	06/Feb/18	wet	Gatamaiyu	Tea40M	B	5	0	0	0	0	0	0	40	
2018	06/Feb/18	wet	Gatamaiyu	Tea40M	B	6	0	0	0	0	0	0	40	
2018	06/Feb/18	wet	Gatamaiyu	Tea40M	B	7	0	0	0	0	0	0	40	
2018	06/Feb/18	wet	Gatamaiyu	Tea40M	B	8	0	0	0	0	0	0	40	
2018	06/Feb/18	wet	Gatamaiyu	Tea40M	B	9	0	0	0	0	0	0	40	
2018	06/Feb/18	wet	Gatamaiyu	Tea40M	B	10	0	0	0	0	0	0	40	
2019	9-Oct-19	dry	Kakamega	Forest	A	1	1	0	1	0	0	0	Forest	Bird
2019	9-Oct-19	dry	Kakamega	Forest	A	2	1	1	0	0	0	0	Forest	Insect
2019	9-Oct-19	dry	Kakamega	Forest	A	3	1	1	0	0	0	0	Forest	Insect
2019	9-Oct-19	dry	Kakamega	Forest	A	4	1	0	1	0	0	0	Forest	Bird
2019	9-Oct-19	dry	Kakamega	Forest	A	5	0	0	0	0	0	0	Forest	
2019	9-Oct-19	dry	Kakamega	Forest	A	6	1	0	1	0	0	0	Forest	Bird
2019	9-Oct-19	dry	Kakamega	Forest	A	7	1	0	1	0	0	0	Forest	Bird
2019	9-Oct-19	dry	Kakamega	Forest	A	8	0	0	0	0	0	0	Forest	
2019	9-Oct-19	dry	Kakamega	Forest	A	9	0	0	0	0	0	0	Forest	
2019	9-Oct-19	dry	Kakamega	Forest	A	10	0	0	0	0	0	0	Forest	
2019	9-Oct-19	dry	Kakamega	Edge	A	1	1	1	0	0	0	0	0	Insect
2019	9-Oct-19	dry	Kakamega	Edge	A	2	0	0	0	0	0	0	0	
2019	9-Oct-19	dry	Kakamega	Edge	A	3	1	1	0	0	0	0	0	Insect
2019	9-Oct-19	dry	Kakamega	Edge	A	4	1	1	0	0	0	0	0	Insect
2019	9-Oct-19	dry	Kakamega	Edge	A	5	0	0	0	0	0	0	0	
2019	9-Oct-19	dry	Kakamega	Edge	A	6	1	0	1	0	0	0	0	Bird
2019	9-Oct-19	dry	Kakamega	Edge	A	7	0	0	0	0	0	0	0	
2019	9-Oct-19	dry	Kakamega	Edge	A	8	0	0	0	0	0	0	0	
2019	9-Oct-19	dry	Kakamega	Edge	A	9	0	0	0	0	0	0	0	

2019	9-Oct-19	dry	Kakamega	Edge	A	10	Lost	0	0	0	0	1	0	Lost
2019	9-Oct-19	dry	Kakamega	Tea5M	A	1	0	0	0	0	0	0	5	
2019	9-Oct-19	dry	Kakamega	Tea5M	A	2	1	1	0	0	0	0	5	Insect
2019	9-Oct-19	dry	Kakamega	Tea5M	A	3	0	0	0	0	0	0	5	
2019	9-Oct-19	dry	Kakamega	Tea5M	A	4	1	1	0	0	0	0	5	Insect
2019	9-Oct-19	dry	Kakamega	Tea5M	A	5	0	0	0	0	0	0	5	
2019	9-Oct-19	dry	Kakamega	Tea5M	A	6	0	0	0	0	0	0	5	
2019	9-Oct-19	dry	Kakamega	Tea5M	A	7	0	0	0	0	0	0	5	
2019	9-Oct-19	dry	Kakamega	Tea5M	A	8	0	0	0	0	0	0	5	
2019	9-Oct-19	dry	Kakamega	Tea5M	A	9	0	0	0	0	0	0	5	
2019	9-Oct-19	dry	Kakamega	Tea5M	A	10	0	0	0	0	0	0	5	
2019	9-Oct-19	dry	Kakamega	Tea20M	A	1	0	0	0	0	0	0	20	
2019	9-Oct-19	dry	Kakamega	Tea20M	A	2	0	0	0	0	0	0	20	
2019	9-Oct-19	dry	Kakamega	Tea20M	A	3	1	1	0	0	0	0	20	Insect
2019	9-Oct-19	dry	Kakamega	Tea20M	A	4	0	0	0	0	0	0	20	
2019	9-Oct-19	dry	Kakamega	Tea20M	A	5	1	1	0	0	0	0	20	Insect
2019	9-Oct-19	dry	Kakamega	Tea20M	A	6	1	1	0	0	0	0	20	Insect
2019	9-Oct-19	dry	Kakamega	Tea20M	A	7	0	0	0	0	0	0	20	
2019	9-Oct-19	dry	Kakamega	Tea20M	A	8	0	0	0	0	0	0	20	
2019	9-Oct-19	dry	Kakamega	Tea20M	A	9	0	0	0	0	0	0	20	
2019	9-Oct-19	dry	Kakamega	Tea20M	A	10	0	0	0	0	0	0	20	
2019	9-Oct-19	dry	Kakamega	Tea40M	A	1	0	0	0	0	0	0	40	
2019	9-Oct-19	dry	Kakamega	Tea40M	A	2	1	1	0	0	0	0	40	Insect
2019	9-Oct-19	dry	Kakamega	Tea40M	A	3	0	0	0	0	0	0	40	
2019	9-Oct-19	dry	Kakamega	Tea40M	A	4	0	0	0	0	0	0	40	
2019	9-Oct-19	dry	Kakamega	Tea40M	A	5	1	1	0	0	0	0	40	Insect
2019	9-Oct-19	dry	Kakamega	Tea40M	A	6	0	0	0	0	0	0	40	
2019	9-Oct-19	dry	Kakamega	Tea40M	A	7	0	0	0	0	0	0	40	
2019	9-Oct-19	dry	Kakamega	Tea40M	A	8	0	0	0	0	0	0	40	
2019	9-Oct-19	dry	Kakamega	Tea40M	A	9	0	0	0	0	0	0	40	
2019	9-Oct-19	dry	Kakamega	Tea40M	A	10	0	0	0	0	0	0	40	
2019	10-Oct-19	dry	Kakamega	Forest	B	1	0	0	0	0	0	0	Forest	
2019	10-Oct-19	dry	Kakamega	Forest	B	2	1	1	0	0	0	0	Forest	Insect
2019	10-Oct-19	dry	Kakamega	Forest	B	3	1	1	0	0	0	0	Forest	Insect
2019	10-Oct-19	dry	Kakamega	Forest	B	4	1	0	1	0	0	0	Forest	Bird
2019	10-Oct-19	dry	Kakamega	Forest	B	5	0	0	0	0	0	0	Forest	
2019	10-Oct-19	dry	Kakamega	Forest	B	6	1	1	0	0	0	0	Forest	Insect
2019	10-Oct-19	dry	Kakamega	Forest	B	7	0	0	0	0	0	0	Forest	
2019	10-Oct-19	dry	Kakamega	Forest	B	8	1	0	1	0	0	0	Forest	Bird
2019	10-Oct-19	dry	Kakamega	Forest	B	9	0	0	0	0	0	0	Forest	
2019	10-Oct-19	dry	Kakamega	Forest	B	10	0	0	0	0	0	0	Forest	
2019	10-Oct-19	dry	Kakamega	Edge	B	1	0	0	0	0	0	0	0	
2019	10-Oct-19	dry	Kakamega	Edge	B	2	1	1	0	0	0	0	0	Insect
2019	10-Oct-19	dry	Kakamega	Edge	B	3	0	0	0	0	0	0	0	
2019	10-Oct-19	dry	Kakamega	Edge	B	4	0	0	0	0	0	0	0	
2019	10-Oct-19	dry	Kakamega	Edge	B	5	1	0	1	0	0	0	0	Bird

2019	10-Oct-19	dry	Kakamega	Edge	B	6	0	0	0	0	0	0	0	
2019	10-Oct-19	dry	Kakamega	Edge	B	7	0	0	0	0	0	0	0	
2019	10-Oct-19	dry	Kakamega	Edge	B	8	0	0	0	0	0	0	0	
2019	10-Oct-19	dry	Kakamega	Edge	B	9	0	0	0	0	0	0	0	
2019	10-Oct-19	dry	Kakamega	Edge	B	10	0	0	0	0	0	0	0	
2019	10-Oct-19	dry	Kakamega	Tea5M	B	1	0	0	0	0	0	0	5	
2019	10-Oct-19	dry	Kakamega	Tea5M	B	2	0	0	0	0	0	0	5	
2019	10-Oct-19	dry	Kakamega	Tea5M	B	3	0	0	0	0	0	0	5	
2019	10-Oct-19	dry	Kakamega	Tea5M	B	4	0	0	0	0	0	0	5	
2019	10-Oct-19	dry	Kakamega	Tea5M	B	5	0	0	0	0	0	0	5	
2019	10-Oct-19	dry	Kakamega	Tea5M	B	6	1	0	1	0	0	0	5	Bird
2019	10-Oct-19	dry	Kakamega	Tea5M	B	7	0	0	0	0	0	0	5	
2019	10-Oct-19	dry	Kakamega	Tea5M	B	8	0	0	0	0	0	0	5	
2019	10-Oct-19	dry	Kakamega	Tea5M	B	9	0	0	0	0	0	0	5	
2019	10-Oct-19	dry	Kakamega	Tea5M	B	10	0	0	0	0	0	0	5	
2019	10-Oct-19	dry	Kakamega	Tea20M	B	1	0	0	0	0	0	0	20	
2019	10-Oct-19	dry	Kakamega	Tea20M	B	2	0	0	0	0	0	0	20	
2019	10-Oct-19	dry	Kakamega	Tea20M	B	3	0	0	0	0	0	0	20	
2019	10-Oct-19	dry	Kakamega	Tea20M	B	4	0	0	0	0	0	0	20	
2019	10-Oct-19	dry	Kakamega	Tea20M	B	5	1	1	0	0	0	0	20	Insect
2019	10-Oct-19	dry	Kakamega	Tea20M	B	6	0	0	0	0	0	0	20	
2019	10-Oct-19	dry	Kakamega	Tea20M	B	7	0	0	0	0	0	0	20	
2019	10-Oct-19	dry	Kakamega	Tea20M	B	8	0	0	0	0	0	0	20	
2019	10-Oct-19	dry	Kakamega	Tea20M	B	9	0	0	0	0	0	0	20	
2019	10-Oct-19	dry	Kakamega	Tea20M	B	10	0	0	0	0	0	0	20	
2019	10-Oct-19	dry	Kakamega	Tea40M	B	1	0	0	0	0	0	0	40	
2019	10-Oct-19	dry	Kakamega	Tea40M	B	2	0	0	0	0	0	0	40	
2019	10-Oct-19	dry	Kakamega	Tea40M	B	3	0	0	0	0	0	0	40	
2019	10-Oct-19	dry	Kakamega	Tea40M	B	4	0	0	0	0	0	0	40	
2019	10-Oct-19	dry	Kakamega	Tea40M	B	5	0	0	0	0	0	0	40	
2019	10-Oct-19	dry	Kakamega	Tea40M	B	6	0	0	0	0	0	0	40	
2019	10-Oct-19	dry	Kakamega	Tea40M	B	7	0	0	0	0	0	0	40	
2019	10-Oct-19	dry	Kakamega	Tea40M	B	8	0	0	0	0	0	0	40	
2019	10-Oct-19	dry	Kakamega	Tea40M	B	9	0	0	0	0	0	0	40	
2019	10-Oct-19	dry	Kakamega	Tea40M	B	10	0	0	0	0	0	0	40	
2019	02/Sep/19	dry	Mau/Kericho	Forest	A	1	1	0	1	0	0	0	Forest	Bird
2019	02/Sep/19	dry	Mau/Kericho	Forest	A	2	0	0	0	0	0	0	Forest	
2019	02/Sep/19	dry	Mau/Kericho	Forest	A	3	1	1	0	0	0	0	Forest	Insect
2019	02/Sep/19	dry	Mau/Kericho	Forest	A	4	1	1	0	0	0	0	Forest	Insect
2019	02/Sep/19	dry	Mau/Kericho	Forest	A	5	0	0	0	0	0	0	Forest	
2019	02/Sep/19	dry	Mau/Kericho	Forest	A	6	0	0	0	0	0	0	Forest	
2019	02/Sep/19	dry	Mau/Kericho	Forest	A	7	1	1	0	0	0	0	Forest	Insect
2019	02/Sep/19	dry	Mau/Kericho	Forest	A	8	1	0	1	0	0	0	Forest	Bird
2019	02/Sep/19	dry	Mau/Kericho	Forest	A	9	1	0	1	0	0	0	Forest	Bird
2019	02/Sep/19	dry	Mau/Kericho	Forest	A	10	0	0	0	0	0	0	Forest	
2019	02/Sep/19	dry	Mau/Kericho	Edge	A	1	1	0	0	1	0	0	0	Mammal

2019	02/Sep/19	dry	Mau/Kericho	Edge	A	2	1	0	1	0	0	0	0	Bird
2019	02/Sep/19	dry	Mau/Kericho	Edge	A	3	1	1	0	0	0	0	0	Insect
2019	02/Sep/19	dry	Mau/Kericho	Edge	A	4	0	0	0	0	0	0	0	
2019	02/Sep/19	dry	Mau/Kericho	Edge	A	5	0	0	0	0	0	0	0	
2019	02/Sep/19	dry	Mau/Kericho	Edge	A	6	0	0	0	0	0	0	0	
2019	02/Sep/19	dry	Mau/Kericho	Edge	A	7	1	1	0	0	0	0	0	Insect
2019	02/Sep/19	dry	Mau/Kericho	Edge	A	8	1	0	1	0	0	0	0	Bird
2019	02/Sep/19	dry	Mau/Kericho	Edge	A	9	1	1	0	0	0	0	0	Insect
2019	02/Sep/19	dry	Mau/Kericho	Edge	A	10	1	1	0	0	0	0	0	Insect
2019	02/Sep/19	dry	Mau/Kericho	Tea5M	A	1	1	0	1	0	0	0	5	Bird
2019	02/Sep/19	dry	Mau/Kericho	Tea5M	A	2	1	0	0	1	0	0	5	Mammal
2019	02/Sep/19	dry	Mau/Kericho	Tea5M	A	3	1	1	0	0	0	0	5	Insect
2019	02/Sep/19	dry	Mau/Kericho	Tea5M	A	4	0	0	0	0	0	0	5	
2019	02/Sep/19	dry	Mau/Kericho	Tea5M	A	5	0	0	0	0	0	0	5	
2019	02/Sep/19	dry	Mau/Kericho	Tea5M	A	6	0	0	0	0	0	0	5	
2019	02/Sep/19	dry	Mau/Kericho	Tea5M	A	7	1	1	0	0	0	0	5	Insect
2019	02/Sep/19	dry	Mau/Kericho	Tea5M	A	8	1	0	1	0	0	0	5	Bird
2019	02/Sep/19	dry	Mau/Kericho	Tea5M	A	9	1	1	0	0	0	0	5	Insect
2019	02/Sep/19	dry	Mau/Kericho	Tea5M	A	10	1	0	1	0	0	0	5	Bird
2019	02/Sep/19	dry	Mau/Kericho	Tea20M	A	1	1	0	0	1	0	0	20	Mammal
2019	02/Sep/19	dry	Mau/Kericho	Tea20M	A	2	0	0	0	0	0	0	20	
2019	02/Sep/19	dry	Mau/Kericho	Tea20M	A	3	0	0	0	0	0	0	20	
2019	02/Sep/19	dry	Mau/Kericho	Tea20M	A	4	1	1	0	0	0	0	20	Insect
2019	02/Sep/19	dry	Mau/Kericho	Tea20M	A	5	0	0	0	0	0	0	20	
2019	02/Sep/19	dry	Mau/Kericho	Tea20M	A	6	0	0	0	0	0	0	20	
2019	02/Sep/19	dry	Mau/Kericho	Tea20M	A	7	1	0	1	0	0	0	20	Bird
2019	02/Sep/19	dry	Mau/Kericho	Tea20M	A	8	1	1	0	0	0	0	20	Insect
2019	02/Sep/19	dry	Mau/Kericho	Tea20M	A	9	0	0	0	0	0	0	20	
2019	02/Sep/19	dry	Mau/Kericho	Tea20M	A	10	0	0	0	0	0	0	20	
2019	02/Sep/19	dry	Mau/Kericho	Tea40M	A	1	1	0	1	0	0	0	40	Bird
2019	02/Sep/19	dry	Mau/Kericho	Tea40M	A	2	0	0	0	0	0	0	40	
2019	02/Sep/19	dry	Mau/Kericho	Tea40M	A	3	0	0	0	0	0	0	40	
2019	02/Sep/19	dry	Mau/Kericho	Tea40M	A	4	1	1	0	0	0	0	40	Insect
2019	02/Sep/19	dry	Mau/Kericho	Tea40M	A	5	0	0	0	0	0	0	40	
2019	02/Sep/19	dry	Mau/Kericho	Tea40M	A	6	0	0	0	0	0	0	40	
2019	02/Sep/19	dry	Mau/Kericho	Tea40M	A	7	0	0	0	0	0	0	40	
2019	02/Sep/19	dry	Mau/Kericho	Tea40M	A	8	0	0	0	0	0	0	40	
2019	02/Sep/19	dry	Mau/Kericho	Tea40M	A	9	0	0	0	0	0	0	40	
2019	02/Sep/19	dry	Mau/Kericho	Tea40M	A	10	0	0	0	0	0	0	40	
2019	04/Sep/19	dry	Mau/Kericho	Forest	B	1	0	0	0	0	0	0	Forest	
2019	04/Sep/19	dry	Mau/Kericho	Forest	B	2	0	0	0	0	0	0	Forest	
2019	04/Sep/19	dry	Mau/Kericho	Forest	B	3	0	0	0	0	0	0	Forest	
2019	04/Sep/19	dry	Mau/Kericho	Forest	B	4	0	0	0	0	0	0	Forest	
2019	04/Sep/19	dry	Mau/Kericho	Forest	B	5	1	0	1	0	0	0	Forest	Bird
2019	04/Sep/19	dry	Mau/Kericho	Forest	B	6	1	0	1	0	0	0	Forest	Bird
2019	04/Sep/19	dry	Mau/Kericho	Forest	B	7	0	0	0	0	0	0	Forest	

2019	04/Sep/19	dry	Mau/Kericho	Forest	B	8	1	0	1	0	0	0	Forest	Bird
2019	04/Sep/19	dry	Mau/Kericho	Forest	B	9	1	0	1	0	0	0	Forest	Bird
2019	04/Sep/19	dry	Mau/Kericho	Forest	B	10	1	0	1	0	0	0	Forest	Bird
2019	04/Sep/19	dry	Mau/Kericho	Edge	B	1	1	0	0	1	0	0	0	Mammal
2019	04/Sep/19	dry	Mau/Kericho	Edge	B	2	1	0	0	1	0	0	0	Mammal
2019	04/Sep/19	dry	Mau/Kericho	Edge	B	3	1	1	0	0	0	0	0	Insect
2019	04/Sep/19	dry	Mau/Kericho	Edge	B	4	1	0	1	0	0	0	0	Bird
2019	04/Sep/19	dry	Mau/Kericho	Edge	B	5	0	0	0	0	0	0	0	
2019	04/Sep/19	dry	Mau/Kericho	Edge	B	6	1	1	0	0	0	0	0	Insect
2019	04/Sep/19	dry	Mau/Kericho	Edge	B	7	1	1	0	0	0	0	0	Insect
2019	04/Sep/19	dry	Mau/Kericho	Edge	B	8	1	0	1	0	0	0	0	Bird
2019	04/Sep/19	dry	Mau/Kericho	Edge	B	9	1	0	1	0	0	0	0	Bird
2019	04/Sep/19	dry	Mau/Kericho	Edge	B	10	1	1	0	0	0	0	0	Insect
2019	04/Sep/19	dry	Mau/Kericho	Tea5M	B	1	1	0	0	1	0	0	5	Mammal
2019	04/Sep/19	dry	Mau/Kericho	Tea5M	B	2	0	0	0	0	0	0	5	
2019	04/Sep/19	dry	Mau/Kericho	Tea5M	B	3	0	0	0	0	0	0	5	
2019	04/Sep/19	dry	Mau/Kericho	Tea5M	B	4	1	0	1	0	0	0	5	Bird
2019	04/Sep/19	dry	Mau/Kericho	Tea5M	B	5	0	0	0	0	0	0	5	
2019	04/Sep/19	dry	Mau/Kericho	Tea5M	B	6	1	1	0	0	0	0	5	Insect
2019	04/Sep/19	dry	Mau/Kericho	Tea5M	B	7	1	0	0	0	0	0	5	Insect
2019	04/Sep/19	dry	Mau/Kericho	Tea5M	B	8	1	0	1	0	0	0	5	Bird
2019	04/Sep/19	dry	Mau/Kericho	Tea5M	B	9	1	1	0	0	0	0	5	Insect
2019	04/Sep/19	dry	Mau/Kericho	Tea5M	B	10	0	0	0	0	0	0	5	
2019	04/Sep/19	dry	Mau/Kericho	Tea20M	B	1	1	0	0	1	0	0	20	Mammal
2019	04/Sep/19	dry	Mau/Kericho	Tea20M	B	2	1	1	0	0	0	0	20	Insect
2019	04/Sep/19	dry	Mau/Kericho	Tea20M	B	3	0	0	0	0	0	0	20	
2019	04/Sep/19	dry	Mau/Kericho	Tea20M	B	4	0	0	0	0	0	0	20	
2019	04/Sep/19	dry	Mau/Kericho	Tea20M	B	5	0	0	0	0	0	0	20	
2019	04/Sep/19	dry	Mau/Kericho	Tea20M	B	6	0	0	0	0	0	0	20	
2019	04/Sep/19	dry	Mau/Kericho	Tea20M	B	7	0	0	0	0	0	0	20	
2019	04/Sep/19	dry	Mau/Kericho	Tea20M	B	8	0	0	0	0	0	0	20	
2019	04/Sep/19	dry	Mau/Kericho	Tea20M	B	9	1	1	0	0	0	0	20	Insect
2019	04/Sep/19	dry	Mau/Kericho	Tea20M	B	10	0	0	0	0	0	0	20	
2019	04/Sep/19	dry	Mau/Kericho	Tea40M	B	1	0	0	0	0	0	0	40	
2019	04/Sep/19	dry	Mau/Kericho	Tea40M	B	2	0	0	0	0	0	0	40	
2019	04/Sep/19	dry	Mau/Kericho	Tea40M	B	3	0	0	0	0	0	0	40	
2019	04/Sep/19	dry	Mau/Kericho	Tea40M	B	4	0	0	0	0	0	0	40	
2019	04/Sep/19	dry	Mau/Kericho	Tea40M	B	5	0	0	0	0	0	0	40	
2019	04/Sep/19	dry	Mau/Kericho	Tea40M	B	6	0	0	0	0	0	0	40	
2019	04/Sep/19	dry	Mau/Kericho	Tea40M	B	7	0	0	0	0	0	0	40	
2019	04/Sep/19	dry	Mau/Kericho	Tea40M	B	8	0	0	0	0	0	0	40	
2019	04/Sep/19	dry	Mau/Kericho	Tea40M	B	9	0	0	0	0	0	0	40	
2019	04/Sep/19	dry	Mau/Kericho	Tea40M	B	10	0	0	0	0	0	0	40	
2019	18/Oct/19	dry	Gatamaiyu	Forest	A	0	0	0	0	0	0	0	Forest	
2019	18/Oct/19	dry	Gatamaiyu	Forest	A	2	0	0	0	0	0	0	Forest	
2019	18/Oct/19	dry	Gatamaiyu	Forest	A	3	0	0	0	0	0	0	Forest	

2019	18/Oct/19	dry	Gatamaiyu	Forest	A	4	0	0	0	0	0	0	Forest	
2019	18/Oct/19	dry	Gatamaiyu	Forest	A	5	0	0	0	0	0	0	Forest	
2019	18/Oct/19	dry	Gatamaiyu	Forest	A	6	0	0	0	0	0	0	Forest	
2019	18/Oct/19	dry	Gatamaiyu	Forest	A	7	0	0	0	0	0	0	Forest	
2019	18/Oct/19	dry	Gatamaiyu	Forest	A	8	1	0	1	0	0	0	Forest	Bird
2019	18/Oct/19	dry	Gatamaiyu	Forest	A	9	1	1	0	0	0	0	Forest	Insect
2019	18/Oct/19	dry	Gatamaiyu	Forest	A	10	1	1	0	0	0	0	Forest	Insect
2019	18/Oct/19	dry	Gatamaiyu	Edge	A	1	0	0	0	0	0	0	0	
2019	18/Oct/19	dry	Gatamaiyu	Edge	A	2	1	1	0	0	0	0	0	Insect
2019	18/Oct/19	dry	Gatamaiyu	Edge	A	3	0	0	0	0	0	0	0	
2019	18/Oct/19	dry	Gatamaiyu	Edge	A	4	1	1	0	0	0	0	0	Insect
2019	18/Oct/19	dry	Gatamaiyu	Edge	A	5	1	1	0	0	0	0	0	Insect
2019	18/Oct/19	dry	Gatamaiyu	Edge	A	6	0	0	0	0	0	0	0	
2019	18/Oct/19	dry	Gatamaiyu	Edge	A	7	0	0	0	0	0	0	0	
2019	18/Oct/19	dry	Gatamaiyu	Edge	A	8	1	1	0	0	0	0	0	Insect
2019	18/Oct/19	dry	Gatamaiyu	Edge	A	9	1	0	1	0	0	0	0	Bird
2019	18/Oct/19	dry	Gatamaiyu	Edge	A	10	0	0	0	0	0	0	0	
2019	18/Oct/19	dry	Gatamaiyu	Tea5M	A	1	0	0	0	0	0	0	5	
2019	18/Oct/19	dry	Gatamaiyu	Tea5M	A	2	1	1	0	0	0	0	5	Insect
2019	18/Oct/19	dry	Gatamaiyu	Tea5M	A	3	0	0	0	0	0	0	5	
2019	18/Oct/19	dry	Gatamaiyu	Tea5M	A	4	0	0	0	0	0	0	5	
2019	18/Oct/19	dry	Gatamaiyu	Tea5M	A	5	0	0	0	0	0	0	5	
2019	18/Oct/19	dry	Gatamaiyu	Tea5M	A	6	1	1	0	0	0	0	5	Insect
2019	18/Oct/19	dry	Gatamaiyu	Tea5M	A	7	1	1	0	0	0	0	5	Insect
2019	18/Oct/19	dry	Gatamaiyu	Tea5M	A	8	1	1	0	0	0	0	5	Insect
2019	18/Oct/19	dry	Gatamaiyu	Tea5M	A	9	0	0	0	0	0	0	5	
2019	18/Oct/19	dry	Gatamaiyu	Tea5M	A	10	0	0	0	0	0	0	5	
2019	18/Oct/19	dry	Gatamaiyu	Tea20M	A	1	0	0	0	0	0	0	20	
2019	18/Oct/19	dry	Gatamaiyu	Tea20M	A	2	0	0	0	0	0	0	20	
2019	18/Oct/19	dry	Gatamaiyu	Tea20M	A	3	0	0	0	0	0	0	20	
2019	18/Oct/19	dry	Gatamaiyu	Tea20M	A	4	0	0	0	0	0	0	20	
2019	18/Oct/19	dry	Gatamaiyu	Tea20M	A	5	0	0	0	0	0	0	20	
2019	18/Oct/19	dry	Gatamaiyu	Tea20M	A	6	0	0	0	0	0	0	20	
2019	18/Oct/19	dry	Gatamaiyu	Tea20M	A	7	1	1	0	0	0	0	20	Insect
2019	18/Oct/19	dry	Gatamaiyu	Tea20M	A	8	1	1	0	0	0	0	20	Insect
2019	18/Oct/19	dry	Gatamaiyu	Tea20M	A	9	0	0	0	0	0	0	20	
2019	18/Oct/19	dry	Gatamaiyu	Tea20M	A	10	0	0	0	0	0	0	20	
2019	18/Oct/19	dry	Gatamaiyu	Tea40M	A	1	0	0	0	0	0	0	40	
2019	18/Oct/19	dry	Gatamaiyu	Tea40M	A	2	0	0	0	0	0	0	40	
2019	18/Oct/19	dry	Gatamaiyu	Tea40M	A	3	0	0	0	0	0	0	40	
2019	18/Oct/19	dry	Gatamaiyu	Tea40M	A	4	0	0	0	0	0	0	40	
2019	18/Oct/19	dry	Gatamaiyu	Tea40M	A	5	0	0	0	0	0	0	40	
2019	18/Oct/19	dry	Gatamaiyu	Tea40M	A	6	0	0	0	0	0	0	40	
2019	18/Oct/19	dry	Gatamaiyu	Tea40M	A	7	0	0	0	0	0	0	40	
2019	18/Oct/19	dry	Gatamaiyu	Tea40M	A	8	0	0	0	0	0	0	40	
2019	18/Oct/19	dry	Gatamaiyu	Tea40M	A	9	0	0	0	0	0	0	40	

2019	18/Oct/19	dry	Gatamaiyu	Tea40M	A	10	0	0	0	0	0	0	40	
2019	19/Oct/19	dry	Gatamaiyu	Forest	B	1	0	0	0	0	0	0	Forest	
2019	19/Oct/19	dry	Gatamaiyu	Forest	B	2	1	1	0	0	0	0	Forest	Insect
2019	19/Oct/19	dry	Gatamaiyu	Forest	B	3	1	0	1	0	0	0	Forest	Bird
2019	19/Oct/19	dry	Gatamaiyu	Forest	B	4	0	0	0	0	0	0	Forest	
2019	19/Oct/19	dry	Gatamaiyu	Forest	B	5	Lost	0	0	0	0	0	Forest	Lost
2019	19/Oct/19	dry	Gatamaiyu	Forest	B	6	1	1	0	0	0	0	Forest	Insect
2019	19/Oct/19	dry	Gatamaiyu	Forest	B	7	1	0	1	0	0	0	Forest	Bird
2019	19/Oct/19	dry	Gatamaiyu	Forest	B	8	1	1	0	0	0	0	Forest	Insect
2019	19/Oct/19	dry	Gatamaiyu	Forest	B	9	0	0	0	0	0	0	Forest	
2019	19/Oct/19	dry	Gatamaiyu	Forest	B	10	1	1	0	0	0	0	Forest	Insect
2019	19/Oct/19	dry	Gatamaiyu	Edge	B	1	0	0	0	0	0	0	0	
2019	19/Oct/19	dry	Gatamaiyu	Edge	B	2	0	0	0	0	0	0	0	
2019	19/Oct/19	dry	Gatamaiyu	Edge	B	3	0	0	0	0	0	0	0	
2019	19/Oct/19	dry	Gatamaiyu	Edge	B	4	1	0	1	0	0	0	0	Bird
2019	19/Oct/19	dry	Gatamaiyu	Edge	B	5	0	0	0	0	0	0	0	
2019	19/Oct/19	dry	Gatamaiyu	Edge	B	6	0	0	0	0	0	0	0	
2019	19/Oct/19	dry	Gatamaiyu	Edge	B	7	1	1	0	0	0	0	0	Insect
2019	19/Oct/19	dry	Gatamaiyu	Edge	B	8	0	0	0	0	0	0	0	
2019	19/Oct/19	dry	Gatamaiyu	Edge	B	9	1	0	1	0	0	0	0	Bird
2019	19/Oct/19	dry	Gatamaiyu	Edge	B	10	1	0	1	0	0	0	0	Bird
2019	19/Oct/19	dry	Gatamaiyu	Tea5M	B	1	0	0	0	0	0	0	5	
2019	19/Oct/19	dry	Gatamaiyu	Tea5M	B	2	0	0	0	0	0	0	5	
2019	19/Oct/19	dry	Gatamaiyu	Tea5M	B	3	0	0	0	0	0	0	5	
2019	19/Oct/19	dry	Gatamaiyu	Tea5M	B	4	0	0	0	0	0	0	5	
2019	19/Oct/19	dry	Gatamaiyu	Tea5M	B	5	0	0	0	0	0	0	5	
2019	19/Oct/19	dry	Gatamaiyu	Tea5M	B	6	0	0	0	0	0	0	5	
2019	19/Oct/19	dry	Gatamaiyu	Tea5M	B	7	0	0	0	0	0	0	5	
2019	19/Oct/19	dry	Gatamaiyu	Tea5M	B	8	1	0	1	0	0	0	5	Bird
2019	19/Oct/19	dry	Gatamaiyu	Tea5M	B	9	0	0	0	0	0	0	5	
2019	19/Oct/19	dry	Gatamaiyu	Tea5M	B	10	1	0	1	0	0	0	5	Bird
2019	19/Oct/19	dry	Gatamaiyu	Tea20M	B	1	0	0	0	0	0	0	20	
2019	19/Oct/19	dry	Gatamaiyu	Tea20M	B	2	0	0	0	0	0	0	20	
2019	19/Oct/19	dry	Gatamaiyu	Tea20M	B	3	0	0	0	0	0	0	20	
2019	19/Oct/19	dry	Gatamaiyu	Tea20M	B	4	0	0	0	0	0	0	20	
2019	19/Oct/19	dry	Gatamaiyu	Tea20M	B	5	0	0	0	0	0	0	20	
2019	19/Oct/19	dry	Gatamaiyu	Tea20M	B	6	0	0	0	0	0	0	20	
2019	19/Oct/19	dry	Gatamaiyu	Tea20M	B	7	0	0	0	0	0	0	20	
2019	19/Oct/19	dry	Gatamaiyu	Tea20M	B	8	0	0	0	0	0	0	20	
2019	19/Oct/19	dry	Gatamaiyu	Tea20M	B	9	1	0	1	0	0	0	20	Bird
2019	19/Oct/19	dry	Gatamaiyu	Tea20M	B	10	1	0	1	0	0	0	20	Bird
2019	19/Oct/19	dry	Gatamaiyu	Tea40M	B	1	0	0	0	0	0	0	40	
2019	19/Oct/19	dry	Gatamaiyu	Tea40M	B	2	1	1	0	0	0	0	40	Insect
2019	19/Oct/19	dry	Gatamaiyu	Tea40M	B	3	1	1	0	0	0	0	40	Insect
2019	19/Oct/19	dry	Gatamaiyu	Tea40M	B	4	1	1	0	0	0	0	40	Insect
2019	19/Oct/19	dry	Gatamaiyu	Tea40M	B	5	0	0	0	0	0	0	40	

2019	19/Oct/19	dry	Gatamaiyu	Tea40M	B	6	0	0	0	0	0	0	40
2019	19/Oct/19	dry	Gatamaiyu	Tea40M	B	7	0	0	0	0	0	0	40
2019	19/Oct/19	dry	Gatamaiyu	Tea40M	B	8	0	0	0	0	0	0	40
2019	19/Oct/19	dry	Gatamaiyu	Tea40M	B	9	0	0	0	0	0	0	40
2019	19/Oct/19	dry	Gatamaiyu	Tea40M	B	10	0	0	0	0	0	0	40
							159	76	78	10	1	1	
